# Bridging Generative AI and Diffusion Models With Molecular Simulation to Design Anti-Quorum-Sensing *De novo* Peptides Targeting LasR of *Pseudomonas aeruginosa*

**DOI:** 10.1007/s12602-026-10956-5

**Published:** 2026-03-14

**Authors:** Abbas Khan, Mona A. Sawali, Anwar Mohammad, Fakhrul Hasssan, Fahad M. Alshabrmi, Eid A. Alatawi, Sergio Crovella, Dong-Qing Wei, Abdelali Agouni

**Affiliations:** 1 Department of Pharmaceutical Sciences, College of Pharmacy, QU Health, Qatar University, P.O. Box 2713, Doha, Qatar; 2 Precision Health Analysis Unit, Translational Research, Dasman Diabetes Institute, Dasman, Kuwait; 3 Faculty of Rehabilitation and Allied Health Sciences, Riphah International University, Islamabad, Pakistan; 4 Department of Medical Laboratories, College of Applied Medical Sciences, Qassim University, Buraydah, 51452 Saudi Arabia; 5 Department of Medical Laboratory Technology, Faculty of Applied Medical Sciences, University of Tabuk, Tabuk, 71491 Saudi Arabia; 6 Department of Biomedical Sciences, College of Health Sciences, QU Health, Qatar University, P.O. Box 2713, Doha, Qatar; 7 Department of Bioinformatics and Biological Statistics, College of Life Sciences and Biotechnology, Shanghai Jiao Tong University, Shanghai, P.R. China

**Keywords:** Pseudomonas aeruginosa, Quorum-sensing, Artificial intelligence, Peptides, LasR

## Abstract

The rapid evolution of generative and diffusion-based method in artificial intelligence (AI) has transformed *de novo* peptide and protein design, enabling the creation of sequence–structure–function relationships beyond conventional heuristic exploration. In this work, we developed an AI-guided molecular design pipeline that integrates diffusion-based generative modeling, inverse folding algorithms, and atomistic simulation to design and evaluate novel peptide inhibitors of the LasR quorum-sensing receptor of *Pseudomonas aeruginosa*. Peptides were generated using multiple complementary AI frameworks, including DiffPepBuilder (a diffusion-based denoising generator), ProteinMPNN (an inverse folding network), RFdiffusion2, Evobind2, and BindCraft (a hybrid sequence–structure generative model). These peptides were then subjected to physicochemical profiling and molecular docking to estimate binding affinities (Kd) and interface complementarity. The top candidates were subjected to 400 ns all-atom molecular dynamics (MD) simulations, performed in triplicate, to assess stability, flexibility, and interaction persistence, and were compared against the experimentally validated peptides benchmark. Post-simulation metrics, including RMSD, RMSF, and radius of gyration (Rg), revealed reproducible but architecture-specific dynamic behaviors, with BindCraft 4 maintaining the most compact and stable configuration, exhibiting minimal fluctuations and consistent binding-site retention. MMGBSA, performed at different time intervals, revealed strong van der Waals and electrostatic stabilization, correlating with sub-micromolar dissociation constants predicted computationally. In contrast, DiffPepBuilder 1–2 exhibited balanced flexibility and moderate stabilization, while ProteinMPNN 2 and the control (Aqs1C) displayed comparatively weaker energetic convergence. Principal Component Analysis (PCA) and Free Energy Landscape (FEL) projections confirmed that BindCraft 4 sampled a single deep energy basin, signifying a thermodynamically optimized conformational state. Collectively, this integrative AI-simulation hybrid computational framework demonstrates the capability of generative diffusion and inverse folding models to design peptides with superior structural stability and binding thermodynamics, offering a rational framework for AI-driven peptide therapeutics targeting *Pseudomonas aeruginosa* communication networks.

**Supplementary information:**

The online version contains supplementary material available at 10.1007/s12602-026-10956-5.

## Introduction

*Pseudomonas aeruginosa* (*P. aeruginosa*) is a Gram-negative opportunistic pathogen with significant adaptability and a major cause of severe hospital-acquired infections (HAIs), especially in intensive care units (ICUs). In fact, it is the most frequently identified etiological cause of ventilator-associated pneumonia (VAP), with attributable mortality rates of 20–50% when infection is caused by multidrug-resistant (MDR) *P. aeruginosa* [1, 2]. *P. aeruginosa* also contributes to progressive lung damage and increased mortality due to chronic airway colonization by the bacterium in cystic fibrosis (CF) patients. The clinical persistence and virulence of this pathogen arise from its remarkable ability to form biofilms, structured, surface-associated microbial communities embedded in a self-produced extracellular polymeric matrix. Biofilms help confer protection from host immune responses and reflect a significant reduction in susceptibility to antibiotic therapy [3].

Despite decades of antimicrobial discovery, treating infections caused by *P. aeruginosa* remains challenging due to its inherent and acquired MDR. Conventional bactericidal treatment often fails because *P. aeruginosa* can evade antibiotic exposure through biofilm and quorum-sensing (QS)-regulated virulence programs [4]. QS is the process by which diffusible signaling molecules, known as autoinducers, mediate regulatory functions in gene expression during biofilm maturation and virulence through the three hierarchical systems las, rhl, and pqs, which control most virulence factors [5]. The master regulator of this hierarchy is LasR, a LuxR-type transcriptional regulator that, upon binding its cognate autoinducer, N-(3-oxododecanoyl)-L-homoserine lactone (3-oxo-C12-HSL), activates hundreds of genes. Structure-function approaches suggest that the ligand-binding domain (LBD) of LasR is flexible, particularly around residues S129 and L130, which are thought to be essential for ligand specificity and activation [6, 7]. Inhibition of LasR by targeting its LBD represents a particularly effective therapeutic strategy, as inhibition at this site disrupts the core signaling pathway underpinning *P. aeruginosa* pathogenicity and disables the downstream virulence hierarchy [7].

The persistent intrusiveness of multidrug-resistant (MDR) *P. aeruginosa* underscores the urgent need for a transition from classical bactericidal treatment to anti-virulence therapy [8]. Targeting QS inhibitors (QSIs) represents a significant pharmaceutical opportunity to reduce the pathogen’s disease-causing potential, restore susceptibility to existing antibiotics, and reduce selective pressure for the development of classical resistance mechanisms [9]. Yet, early investigations of small-molecule QSIs reported significant limitations in their recognition, including a lack of selectivity, poor metabolic stability, and insufficient intracellular accumulation, which could limit their ability to engage putative cytoplasmic targets like LasR [10, 11]. However, peptide-based inhibitors have emerged as attractive alternatives for use as a QSI. Peptides are linear but have defined scaffolds, are structurally well-defined, exhibit pronounced flexibility and molecular selectivity, and engage interaction partners through broadly multipoint binding interfaces [12]. This makes them suitable for blocking dynamic protein-protein interactions, or large, often shallow, ligand-binding pockets, such as the flexible LBD of LasR [6]. Peptides lock the LBD in an inactive conformation by covering a larger surface area. This mechanism not only suppresses virulence but also reduces the likelihood of rapid resistance development that is often observed with smaller, more rigid chemical inhibitors [12, 13].

Recent advancements in artificial intelligence (AI) have revolutionized peptide discovery by enabling the *de novo* generation of peptide sequences, underpinned by structural and energetic precision [14, 15]. Moreover, advancements in deep generative frameworks, such as RFdiffusion, ProteinMPNN, BindCraft, and EvoBind3, utilize diffusion modeling, graph neural networks, and interface-confidence metrics (iPAE, ipTM) to optimize the geometry and affinity of binders [6, 7]. For instance, many therapeutically active peptides that are robust, selective, and experimentally validated across various disease areas illustrate advances in AI-driven peptide design. Chen et al.. combined deep neural and convolutional architectures with site-directed mutagenesis to design a *de novo* GPR40 agonist peptide exhibiting nanomolar activity against type 2 diabetes mellitus, with an R² > 0.8 between predicted and experimental potency [16]. Cho et al. demonstrated the integration of CycleRFDiffusion, ProteinMPNN, and HighFold models to design cyclic peptide binders targeting the microglial receptor TREM2, which were confirmed by biophysical assays, including microscale thermophoresis and surface plasmon resonance [17]. In the infectious-disease space, Jiang et al.. reported an AI-guided AMP-hydrogel platform capable of achieving > 99.99% bactericidal efficacy against MRSA and *E. coli*, in addition to accelerating wound healing in vivo [18]. Additionally, Tian et al. identified two novel antimicrobial peptides by combining generative adversarial network and molecular dynamics (MD) simulations, which were highly active against both Gram-positive and Gram-negative bacteria [19]. In particular, the RFpeptides framework extends RFdiffusion through diffusion-based backbone generation to design macrocyclic peptides, several of which have been experimentally validated with nanomolar binding affinities (Kd < 10 nM) across diverse protein targets. Importantly, multiple designed macrocycle–protein complexes were confirmed by X-ray crystallography, revealing high structural concordance between predicted and experimentally resolved binding modes at atomic resolution [20]. Similarly, the BindCraft generative design platform has demonstrated experimental success in de novo binder discovery, producing nanomolar-affinity binders without reliance on high-throughput screening or iterative experimental optimization, and showing functional effects in biological assays, including reduced IgE binding in patient-derived samples and modulation of CRISPR-Cas9 activity [21, 22]. Thus, AI-based generative models, combined with physics-based validation, offer a streamlined, target-agnostic pipeline for designing stable, selective peptide inhibitors for different targets such as LasR.

Given the clinical potential of these methods, we also employed an AI-guided, generative-physics hybrid pipeline to generate peptide antagonists targeting the LasR LBD. The pipeline workflow consists of AI-guided peptide sequence generation, followed by structural modeling and docking refinement using a state-of-the-art AI scoring function. From the complexes generated, those that achieved high confidence thresholds (iPAE ≤ 0.4) by targeting the critical residues were subjected to 1.2 µs each (400 ns × 3) atomistic simulation, post-simulation trajectory analysis, and binding free energy calculation over different time intervals. The designed peptides were benchmarked against the known inhibitors Aqs1A, Aqs1B, Aqs1C, and WTIAVPGPPHS, representing a next-generation framework for developing stable, selective QS blockers in *P. aeruginosa*.

## Methodology

### Data Retrieval & Target Preparation

The crystal structure of the *P. aeruginosa* LasR Ligand Binding Domain (LBD) was retrieved from the Protein Data Bank (PDB) in complex with the PDB ID 6V7W [23]. This bound complex was used for the identification of ligand-binding cavity and critical residues. The pre-processing protocol involved complex preparation using Maestro (Schrödinger Suite) and ChimeraX software, systematically removing all co-crystallized solvent molecules, non-essential ions, and non-structural ligands [24, 25]. Regions with missing loops or residues, particularly flexible regions near the binding site that are involved in ligand binding, were modeled using the loop modeling module in the Protein Preparation Wizard. Hydrogen atoms were added, and the protonation states of all ionizable residues (Asp, Glu, Lys, Arg, His) were calculated and assigned at physiological pH (7.4). The prepared structure was subjected to a comprehensive geometrical and stereochemical quality assessment to ensure structural integrity, suitability for downstream computational design studies, and adherence to accepted standards for high-resolution structures.

### Benchmarking and Critical Residues Analysis

Due to the limited number of experimentally verified LasR-binding peptides, benchmarking was performed using all four peptides reported in the literature: Aqs1A, Aqs1B, Aqs1C, and WTIAVPGPPHS. These controls were docked to LasR using a standardized HADDOCK 2.4 protocol with identical receptor preparation, AIR definitions, and scoring functions. Their HADDOCK scores, Z-scores, energetic decomposition, and interface BSA established the quantitative performance window for known binders. All designed peptides were then evaluated under the same protocol, enabling direct comparison against this benchmark [13]. Predefined restraints were applied to direct contact formation toward experimentally supported interfaces, improving the biological relevance of predicted interactions. Complexes containing multiple clusters of the highest HADDOCK scores were ranked by HADDOCK score and desolvation energy to minimize false positives. Contact mapping of the highest scoring complexes at the atomic level was visualized using PyMOL and PDBsum to assess intermolecular interaction composition and total contributions of the critical residues [26, 27].

### AI-multimodal Based Peptide Designing and Generation

AI-driven methods, including BindCraft [28], ProteinMPNN [29], DiffPepBuilder [30], RFdiffusion 2 [31], and EvoBind [32], were employed to generate *de novo* peptide sequences targeting the LBD site in LasR. BindCraft generates potentially complementary peptide sequences using a structure-conditioned diffusion model informed by receptor surface electrostatics. This approach assumes that the complementary peptide and receptor can enhance favorable interactions, provided the peptide maintains overall shape complementarity with the binding interface. ProteinMPNN uses a graph neural network to optimize amino acid sequences given a fixed backbone geometry. DiffPepBuilder and RFdiffusion 2 both leverage diffusion-based denoising methodologies in 3D coordinate space to iteratively build custom peptide structures conditioned on receptor topology. Both methods allow iterative construction of peptide backbones that adopt metabolic conformations while remaining complementary to the receptor interface. EvoBind3 combines an evolutionary sequence prior with a diffusion-based structural refinement, enabling co-optimization of peptide stability and receptor binding. The binding interface was spatially defined as a 4 Å radius centered on the crystallographic binding site of the natural autoinducer 3-oxo-C12-HSL. Peptide lengths were restricted to 16–20 amino acids, corresponding to typical standard ranges used for synthetically accessible and structurally stable peptides.

### Physicochemical Landscape of Designed Peptides

All generated sequences were subjected to rigorous physicochemical properties evaluation to ensure optimal synthetic feasibility, stability, and potential cellular activity, given that LasR functions within the cytoplasmic environment of *P. aeruginosa* using Biopython’s ProteinAnalysis module (*Bio.SeqUtils.ProtParam*). The calculated physiochemical properties include molecular weight, isoelectric point (pI), charge at physiological pH, hydrophobicity (GRAVY), aromaticity, instability index, aliphatic index, extinction coefficient, and fractions of secondary structures, applying the prescribed models for calculating hydrophobicity (Kyte–Doolittle, 1982), as well as the models of Bjellqvist et al. (1993), Guruprasad et al. (1990), Ikai (1980), Pace et al. (1995), and Chou–Fasman (1974) [33–36]. The amino acid composition was further categorized based on charged, polar, and hydrophobic groups to evaluate polarity, stability, and folding tendencies. In addition, each sequence was screened using predictive aggregation-propensity models. Peptides potentially susceptible to fibrillation or aggregation, particularly under conditions affected by counterion concentration, were identified and excluded, using the Aggrescan4D (A4D) server (https://biocomp.chem.uw.edu.pl/a4d/) [35, 37]. Data analysis and statistical comparisons were performed using Pandas and NumPy, and visualizations were created through Matplotlib and Seaborn.

### Structure Modeling & Folding

High-priority peptide candidates were advanced to complex structure prediction using AlphaFold3 (AF3) and other complementary high-accuracy folding frameworks, such as ESMFold and PyRosetta [28, 38–40]. Among the AI-driven peptide design frameworks used, RFDiffusion 2, DiffPepBuilder, and BindCraft all embed PyRosetta natively as their structural backend, responsible for coordinate changes, energy calculations, and relaxation during sequence–structure optimization. By contrast, ProteinMPNN and EvoBind3 focus on generating sequences and coarse peptide geometries via diffusion- or inverse-folding and lack a structure-prediction module. AF3 was utilized to generate the three-dimensional conformation of each peptide in a single predictive pass, and the models were evaluated using the Predicted Local Distance Difference Test (pLDDT) score across which interfacial residues were required to be ≥ 70, reflecting high confidence in local backbone geometry [28]. Furthermore, the average interface Predicted Aligned Error (iPAE) was constrained to ≤ 0.4, providing a quantitative measure of geometric precision at the intermolecular binding interface [36]. All predicted complex models were subsequently refined through energy minimization using the AMBER ff19SB force field [41]. This approach resolved local conformational strain and removed any high-energy van der Waals clashes resulting from the initial prediction, and established a relaxed, energetically stable geometry for downstream computational analyses.

### Integrated Physics and AI-Enabled Protein-Peptide Docking

The engineered peptides were evaluated using physics-based and AI-enabled docking methods to assess the potential binding landscape and the robustness of the structures developed. A standard physics-based mode of docking, such as HADDOCK, was also included for the initial scoring of our designed peptides, followed by several AI-based approaches, such as DiffPepdock and Boltz2, that address challenges in HADDOCK and yield higher rates of predicting protein-short peptide complexes. DiffPepdock uses SE(3)-equivariant diffusion models, and Boltz2 quickly samples a large ensemble of potential binding poses for each candidate peptide [42–44]. The centroid structures from the three largest clusters were selected for the subsequent step. This facilitates MD simulations in capturing several diverse yet relevant binding modes, adequately sampling conformational space to prevent bias towards a single kinetically trapped pose and providing a more reliable thermodynamic estimate of peptide-target interactions.

### Peptides Ranking and Affinity Evaluation Through Dissociation Constant (K_D_)

To explicitly rank and compare the designed peptide candidates under identical physiological and structural conditions, we used PRODIGY (PROtein binDing enerGY prediction) to determine the comparative binding strength rather than as absolute measures of experimental binding affinity [45]. PRODIGY estimates the binding affinity of molecules based on their structural contact features. The training and validation for PRODIGY is performed using large sets of experimentally solved complexes, from which the estimated binding affinities have been compared with those from experiments and resulted in a Pearson correlation coefficient of approximately 0.73 and a root mean square error (RMSE) of approximately 1.89 kcal/mol in benchmark testing. Therefore, PRODIGY was used to estimate and rank the complexes generated by docking protocols.

### Atomistic Molecular Dynamics (MD) Simulations

MD simulations were executed using the Amber suite. System preparation and parameters of the simulations were established according to the protein-peptide complex to maximize accuracy and comprehensive conformational sampling of the system of interest, and therefore represent a relevant description of the peptide–LasR complex under physiological conditions. The FF19SB force field was used to parameterize the protein–peptide complexes [46]. Each system was then solvated in a periodic octahedral box filled with explicit OPC water molecules, ensuring a 12 Å buffer between the solute and the box boundaries [46]. To achieve electrostatic neutrality, appropriate counterions were added, and the ionic environment was adjusted to physiological strength. We performed restrained and full minimization for each complex. This initial step relaxed initial steric clashes and equilibrated solvent molecules around the crystal-derived solute structure. The system underwent 5,000 steps of steepest descent followed by 5,000 steps of conjugate gradient minimization, applying harmonic restraints (50 kcal·mol⁻¹·Å⁻²) on solute heavy atoms. A final 10,000 steps of unrestrained conjugate gradient minimization were performed for the entire system to relieve remaining local high-energy contacts. Thereafter, each system was gradually heated from 0 K to 300 K over 500 ps in the canonical (NVT) ensemble, while maintaining weak restraints on Cα atoms using the Langevin thermostat (γ = 1.0 ps − 1).

In comparison, an unrestrained 500 ps equilibration was then conducted under isothermal–isobaric (NPT) conditions at 300 K and 1 bar, employing the Monte Carlo barostat for pressure regulation [47]. Finally, for each complex, three independent 400 ns production simulations were run, yielding a total of 1.2 µs of sampling per peptide system. Simulations were conducted in the NPT ensemble with periodic boundary conditions, employing a 2-fs time step, Particle Mesh Ewald (PME) for long-range electrostatics, and SHAKE constraints on all bonds involving hydrogen atoms. The extended 400ns duration was essential to capture slow conformational transitions within the flexible LasR LBD binding loop [6], thereby mitigating kinetic trapping and providing a thermodynamically representative ensemble for binding free energy calculations [48].

### Trajectory Analysis and Dynamic Metrics

MD trajectories were analyzed using the CPPTRAJ module of AMBER24, and supplemental, more advanced statistical post-processing was performed with Python 3.12 (NumPy, Pandas, SciPy, Matplotlib). After equilibration, all trajectories were used for the post-simulation processing. The averages and statistical properties are presented using three independent replicas. The results are explored for reproducibility and convergence reliability [44]. Root Mean Square Deviation (RMSD), Radius of Gyration (Rg), and Root Mean Square Fluctuation (RMSF) were computed using the CPPTRAJ approach of AMBER24 to quantify structural stability, compactness, and residue-level dynamics of three independent replicas. The RMSD was evaluated considering the heavy atoms of the peptide and the backbone of the protein to the minimized starting structure, using the ensemble mean (µ) and standard deviations (σ), where all frames collected from equilibration were averaged to determine stability. The assessment of Rg was performed using the radgyr command to determine the complex’s global compactness over time. Residual RMSF was procured from the Cα atoms with values normalized to the average backbone mean-square deviation to allow for inter-complex comparison. RMSF profiles were averaged over the three replicas to attain means ± SD, revealing that local peaks were coincident with flexible loop or solvent-exposed regions and local depressions corresponded to rigid, anchored residues in the binding interface topology. Statistical significance was determined using a Kruskal–Wallis test (H = 14,139.725, *p* < 0.0001) on pooled RMSF distributions to assess differences across all peptides. Empirical Cumulative Distribution Functions (ECDFs) described the fraction of the residue with either insignificant or substantial motion amplitude. Strong intra-replica agreements (*r* = 0.86–0.98) were obtained from Pearson inter-replica correlation of residue-wise RMSF vectors, revealing concrete support for reproducibility and moderate agreement across peptides (*r* = 0.68–0.79).

### Conformational Sampling Assessment and Convergence

To verify that the 400 ns simulations had sufficiently explored the essential conformational space, two primary convergence analyses were performed [48].

#### Principal Component Analysis (PCA)

Principal component analysis (PCA) was performed on the Cα atomic coordinates of the LasR–peptide complexes to decompose the overall molecular motion into a smaller set of dominant collective modes (PC1, PC2). Inspection of the three simulation replicates indicated that these principal motions were consistently sampled across replicates, supporting convergence and reproducibility of the dynamics [48].

#### Free Energy Landscape (FEL)

The free energy landscape (FEL) was developed by projecting the sampled conformations onto the first two principal components (PC1 and PC2) to map the population density and identify the lowest-energy basin corresponding to the most thermodynamically stable bound state of the complex. These approaches have been used to explore the conformational changes in various biological systems [49, 50].

### Binding Affinity & Dissociation Constant Predictions

The Molecular Mechanics/Generalized Born Surface Area (MM-GBSA) method was employed to estimate the binding free energy of the LasR LBD-peptide complexes [51]. This endpoint-free energy method employs conformational ensembles derived from production-phase MD trajectories, providing greater accuracy than conventional scoring functions by explicitly accounting for solvent interactions and thermal fluctuations [51]. For each system, the first and last 10ns snapshots were uniformly extracted from each of the three independent production MD replicates, to calculate the binding free energy, ensuring a statistically robust and reliable protocol. MM-GBSA calculations were employed for comparative evaluation of changes in binding energetics using a consistent solvation model across all systems, recognizing that absolute free energies are method-dependent and best interpreted in a relative context. The binding free energy is estimated using the following thermodynamic equation:

$$\begin{aligned}&\:{\varDelta\:G}_{binding}={G}_{complex}-\left({G}_{protein}+{G}_{ligand}\right)\\&\approx\:\varDelta\:EMM+\:{\varDelta\:G}_{solvation} \end{aligned}$$

Each component is calculated as follows:

- ΔE_MM_= Represents the change in molecular mechanical energy between the bound complex [51].
- ΔG_solvation_​: The change in solvation free energy upon complex formation, composed of polar ΔG_polar_ and non-polar ΔG_non−polar_ terms.

Standard Errors of the Mean (SEM) for ΔG_binding​_ values were determined by calculating the average and standard deviation across the three independent MD replicates, providing crucial confidence intervals for the statistical validation of binding affinity [51].

### Visualization, Ensemble Averaging, and Statistical Analysis

All plots, including RMSD, Rg, RMSF line graphs, violin plots, ECDF curves, and Pearson correlation heatmaps, were generated using Matplotlib (v3.9). Violin plots depict the density and median spread of RMSF distributions, while ECDFs illustrate cumulative stability behavior. Across replicas for each peptide, Kruskal–Wallis nonparametric tests were applied to determine whether the distributions originated from the same population. The combined statistical and graphical treatment allowed quantification of stability, per-residue flexibility, global rigidity, and reproducibility with high precision.

## Results and Discussion

### Analysis of the Control Peptides and Benchmarking

**Fig. 1:**
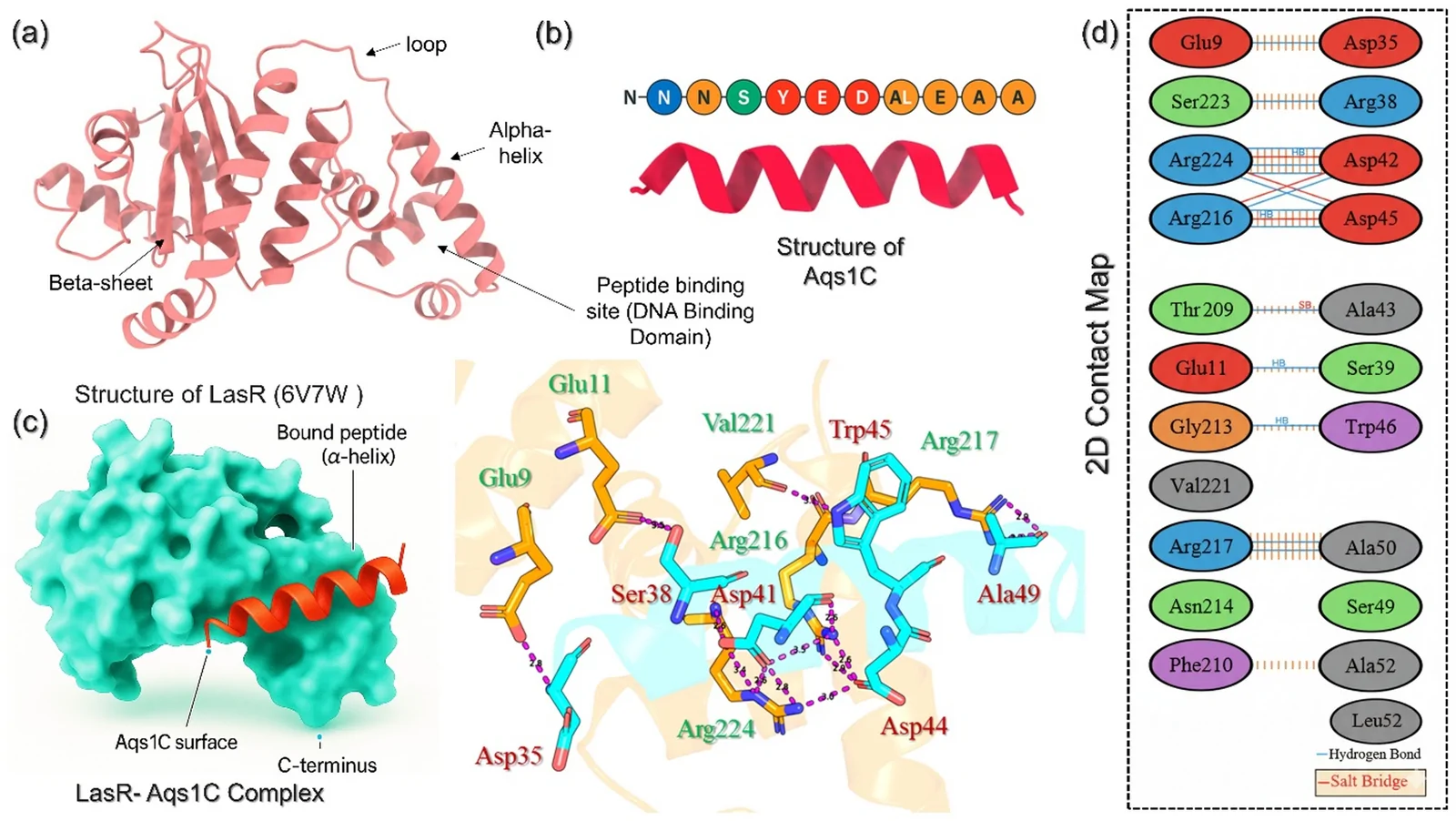
Structural and interaction analysis of the LasR–Aqs1C complex. (**a**) Cartoon representation of the structure of the LasR transcriptional regulator (PDB ID: 6V7W), showing the peptide-binding pocket within the DNA-binding domain and distribution of different secondary structural elements. (**b**) Cartoon representation of the α-helical Aqs1C peptide, with individual residues color-coded. (**c**) LasR bound to Aqs1C, as surface representation, and the detailed 3D interactions interface showing the key contacts. (**d**) 2D schematic contact map highlighting the key hydrogen bonds (blue dashed lines) and salt bridges (red dashed lines) established between Aqs1C and LasR residues

To establish a reference binding baseline for downstream peptide evaluation, all four reported LasR-binding peptides (Aqs1A, Aqs1B, Aqs1C, and WTIAVPGPPHS) were docked. HADDOCK generated four validated control peptide complexes to determine the binding of these control peptides with the LasR-binding cavity. WTIAVPGPPHS (control 1) displayed a HADDOCK score of **− 71.8 ± 1.8** kcal/mol with a favorable van der Waals term **(− 53.6 ± 2.0)** and moderate electrostatic contribution **(− 41.0 ± 7.8)**, yielding a Z-score of − 2.4, the most favorable among controls. Aqs1A exhibited the strongest electrostatic energy **(− 228.1 ± 21.4)** and overall, HADDOCK score **(− 81.2 ± 5.6)**, though with a smaller cluster size and higher restraint violation, consistent with a more polar binding mode. Aqs1B showed intermediate energetics (HADDOCK score **− 79.7 ± 5.5**) but reduced convergence (RMSD4.6 ± 0.4) and a modest Z-score (− 1.7). Finally, the structural analysis of the LasR–Aqs1C complex reveals a cooperative network of electrostatic, hydrogen-bonding, and hydrophobic interactions that govern peptide recognition and QS inhibition. The Aqs1C peptide adopts a stable α-helical conformation that inserts into the DNA-binding domain of LasR, sterically occupying the interface required for transcriptional regulation. The control achieved a HADDOCK score of **− 83.1 ± 2.8** kcal/mol, supported primarily by van der Waals packing (E_vdW_ **−42.8 ± 5.9** kcal/mol) and moderate electrostatics (E_elec_ **−215.3 ± 24.2** kcal/mol). It maintained multiple hydrogen bonds and salt bridges across the interface. Among the hydrogen bonds, Glu9-Asp35, Arg216-Asp45, Arg216-Asp42, Arg217-Ala50 (2.85Å), Arg224-Asp42, and Arg224-Asp45 (3.04Å) were reported, while at the core of this binding event, Asp42 and Asp45 from the peptide form persistent salt bridges with LasR Arg216 and Arg224, respectively, anchoring the helix deeply within the pocket. Similarly, the acidic residues, including Glu9 and Glu11, interact reciprocally with the peptide’s basic and polar residues, thereby reinforcing the electrostatic landscape. Importantly, mutational and biochemical studies have confirmed the functional relevance of several of these residues in LasR activity. Arg216 and Arg224 are consistently identified as critical determinants of ligand recognition and stability within the LasR DNA-binding domain [13]. Similarly, Arg216 and Arg224 have been identified as major contributors to maintaining a robust hydrogen-bond/salt-bridge network [12]. The cooperative action of these residues explains the high-affinity binding of Aqs1C and supports the concept that disruption of any of these electrostatic pairs severely compromises inhibitory potential. Quantitative comparison of HADDOCK scores, Z-scores, energy decomposition profiles, and interface burial revealed that Aqs1C consistently outperformed all other control peptides, representing the highest-affinity interaction within the validated peptide set. Aqs1C achieved the most favorable HADDOCK score (− 83.1 ± 2.8 kcal/mol), exceeding the docking performance of Aqs1A, Aqs1B, and WTIAVPGPPHS in both magnitude and consistency. This superior energetic profile made Aqs1C the most appropriate benchmark for subsequent comparative analyses and the primary reference for evaluating designed peptides. Collectively, these insights provide a rational foundation for the *de novo* design of LasR-targeting peptides by treating Arg216 and Arg224 as key hotspots for the new binders. We therefore considered the essential hotspots in LasR, such as Arg216 and Arg224, to design next-generation inhibitors with enhanced affinity and stability. Such engineered peptides offer a promising avenue for disrupting QS in *P. aeruginosa* and have potential therapeutic relevance for combating multidrug-resistant infections. The crystallographic structure of LasR and the native peptide are given in Fig. 1a and b. The detailed 3D and 2D contact maps are shown in Fig. 1c and d, respectively.

### Multi-Model AI Framework for *de novo* Peptide Design

**Fig. 2:**
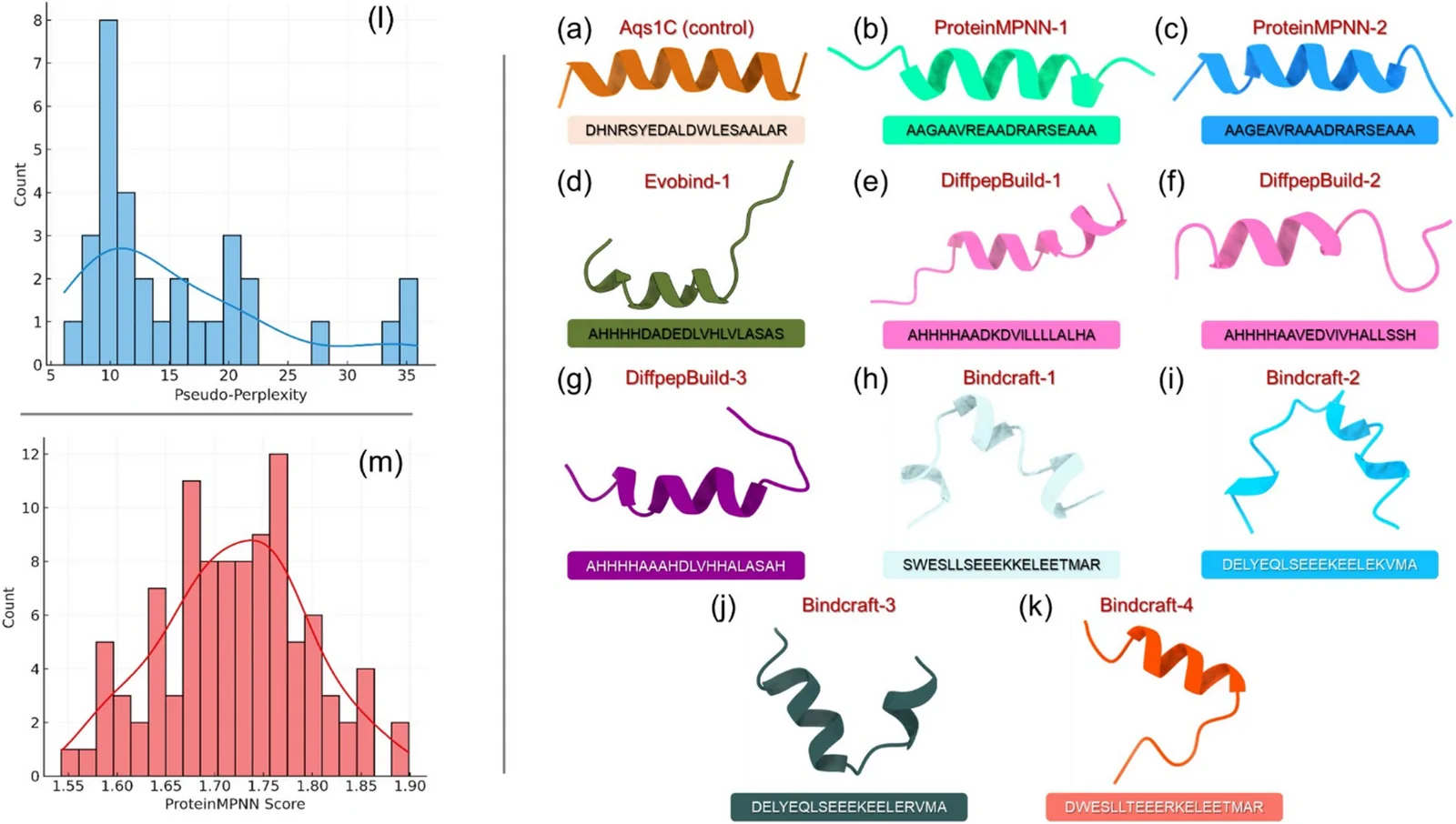
3D structural coordinates and distribution of pseudo-perplexity and sequence likelihood scores. (**a**) Shows the control peptide (Aqs1c), (**b**-**c**) Peptides designed from ProteinMPNN, (**c**) Peptide structure and sequence from Evobind2, (**e**-**g**) shows the peptides designed by DiffPepBuilder, while (**e**-**g**) shows the peptides designed by BindCraft

We identified the critical binding determinants, including Arg216 and Arg224, reported as central binding hotspots to guide *de novo* peptide binder design against the LasR DNA-binding domain using five state-of-the-art artificial intelligence algorithms: BindCraft, DiffPepBuilder, ProteinMPNN, RFdiffusion 2, and EvoBind 2. A JSON input file specifying these key residues and additional geometric constraints served as the design scaffold, allowing each model to explore the sequence–structure landscape around those binding regions. Each model employs a unique algorithm-based approach and together provides independent sampling of the conformational, energetic, and evolutionary subspaces. The structure of the control peptide is shown in Fig. 2a. ProteinMPNN was employed, which reported 100 unique sequences with likelihood scores ranging from 0 to 100 (mean ≈ 17.4). The two highest-scoring sequences (> 90) provided sufficient energetic coherence and geometric validity. These two peptides, based on the likelihood score, were selected for the subsequent structure modeling, *AAGAAVREAADRARSEAAA* and *AAGEAVRAAADRARSEAAA*, and analysis as shown in Fig. 2b and c. Moreover, in parallel, RFdiffusion 2 further enhanced conformational sampling via rigid-frame sampling, and EvoBind2 employed a pseudo-likelihood measure for evolutionary filtering, focusing on retaining stability and mutation tolerance through sampled mutations **(**Fig. 2d**)**. On the other hand, DiffPepBuilder, another diffusion-based independent approach, generated 32 candidates, organized by pseudo-perplexity score, a probabilistic measure of uncertainty related to diffusion certainty, with an average score of 15.65 (range: 6.14–35.92). Among the 32 designs, only three peptide sequences (*AHHHHAADKDVILLLLALHA*,* AHHHHAAVEDVIVHALLSSH*, and *AHHHHAAAHDLVHHALASAH*) **(**Fig. 2e and g**)** with pseudo-perplexity scores < 10 exhibited low-entropy folds with distinctly low uncertainty and were selected for the modeling and downstream processing. BindCraft uses a reinforcement-learning-driven model of molecular diffusion and completed 702 trajectories to design and sample a variety of stochastic peptide–receptor conformational models. Convergence analysis detected four high-confidence binders (*SWESLLSEEEKKELEETMAR*,* DELYEQLSEEEKEELEKVMA*,* DELYEQLSEEEKEELERVMA*, and *DWESLLTEEERKELEETMAR*) **(**Fig. 2h and k**)**, with energetic (< -8 kcal·mol⁻¹) and geometric convergence (= 1.0) confirming the validity and conformational feasibility of these peptides. Thus, based on the validated criteria, these peptides were selected for further processing and analysis. In total, the independent generative frameworks collectively converged on an ensemble of LasR-binding peptides, demonstrating the potential of AI-driven multimodal design to support independent peptide hypotheses in high-dimensional conformational space. The pseudo-perplexity distribution (32 peptides) generated by DiffPepBuilder and the ProteinMPNN sequence-likelihood score distribution (100 peptides) are provided in Fig. 2l and m. The finalized peptide ensemble served as the foundation for subsequent AI-accelerated docking and atomistic simulation analyses, enabling rigorous assessment of receptor compatibility, interfacial stability, and binding energetics within the LasR LBD. These validated designs thus transitioned from in silico generation to detailed structural and thermodynamic evaluation.

### Docking of LasR With the de Novo Designed Peptides

The designed peptides were either modeled based on the integral function or docked using external AI-based docking tools. Among the structural modeling, PyRosseta, ESMfold2, and AlphaFold3 were used to generate and refine the 3D structural coordinates for docking. The docking results revealed significant energetic differences between the control peptide (Aqs1C) and the AI-designed peptides, reflected in their HADDOCK, vdW, and electrostatic energy rankings, and were revalidated via DiffPepdock. The control Aqs1C peptide had an average HADDOCK score of **-83.1 ± 2.8 kcal/mol**, which served as the baseline for comparison. The peptides designed by DiffPepBuilder were consistently ranked higher than Aqs1C, especially DiffPepBuilder 1 **(-87.3 ± 3.6 kcal/mol)** and DiffPepBuilder 3 **(-88.3 ± 3.2 kcal/mol)**, which had lower HADDOCK scores, and greater energetic vdW terms − 41.30 and − 45.20 kcal/mol, respectively. This shows a higher binding complementarity between DiffPepBuilder 1 and DiffPepBuilder 3 relative to Aqs1C. Moreover, DiffPepBuilder 2 reported a HADDOCK score of **-85.7 ± 3.3 kcal/mol**, with the total electrostatic energy of -161.4 ± 17.7 kcal/mol, and was comparatively higher than the two DiffPepBuilder peptides 1 and 3, indicating that less charge complementarity was occurring with Aqs1C than with DiffPepBuilder 1 and DiffPepBuilder 3. The EvoBind 1 complex reported a docking score of **-83.4 ± 0.9 kcal/mol**, which was almost identical to that of Aqs1C (control). The vdW and electrostatic terms were less favorable than those in Aqs1C. This suggests that the EvoBind 1 complex was less stable at the interface but may still exhibit promise for experimental testing. In addition, the ProteinMPNN designs reported mixed results, such as ProteinMPNN 1 had a higher (less favorable) HADDOCK score **− 66.0 ± 8.1 kcal/mol**, but ProteinMPNN 2 had a strong binding profile of **-88.2 ± 1.2 kcal/mol**, which was additionally supported by an exceptionally high electrostatic energy of **-338.2 ± 21.4 kcal/mol** and thus indicate tight binding steered by electrostatic anchoring. The BindCraft series produced variable docking results. BindCraft 4 reported a docking score of **-94.8 ± 4.9 kcal/mol**, which had the strongest HADDOCK score among these, with the most favorable electrostatic energy (-350.7 ± 42.0 kcal/mol) and relatively balanced vdW energetic terms. The findings from BindCraft 4 strongly indicated significant polar and non-polar complementarity within the LasR binding pocket. Overall, based on the HADDOCK scores, the designed peptides can be ranked as BindCraft 4, DiffPepBuilder 3, DiffPepBuilder 2, ProteinMPNN 2, and DiffPepBuilder 1, as confirmed by DiffPepdock. The DiffpepDock and Boltz-2 also produced the high cond. These peptides were selected for subsequent analysis, and the remaining peptides were discarded. The docking scores and other parameters are given in Table 1.

**Table 1:** Ranking of the designed peptides based on the docking scores

Parameters	HADDOCK score	Cluster size	RMSD from the overall lowest-energy structure	Van der Waals energy	Electrostatic energy	Desolvation energy	Restraints violation energy	Buried Surface Area	Z-Score
Aqs1C (control)	-83.1+/-2.8	45	10.3+/-16.7	-41.5+/-1.2	-212.7+/-26.2	-10.0+/-1.4	110.0+/-22.9	1170.1+/-46.5	-2.2
DiffPepBuilder 1	-87.3+/-3.6	18	0.3+/-0.2	-41.3+/-1.8	-237.7+/-5.3	-9.3+/-2.9	108.3+/-13.9	1350.8+/-57.9	-1.5
DiffPepBuilder 2	-85.7+/-3.3	32	0.4+/-0.3	-42.0+/-3.3	-161.4+/-17.7	-19.3+/-3.1	78.7+/-38.6	1247.1+/-36.6	-1.8
DiffPepBuilder 3	-88.3+/-3.2	68	1.4+/-0.9	-45.2+/-6.8	-125.0+/-13.2	-23.5+/-3.9	54.2+/-24.5	1264.5+/-79.6	-1.7
Evo Bind 1	-83.4+/-0.9	20	5.5+/-0.3	-38.9+/-0.8	-172.7+/-9.6	-18.0+/-2.5	79.5+/-15.7	1155.5+/-57.0	-1.9
ProteinMPNN 1	-66.0+/-8.1	5	0.4+/-0.3	-40.8+/-5.0	-156.0+/-14.7	1.0+/-2.9	50.9+/-15.2	1224.5+/-60.3	-1.3
ProteinMPNN 2	-88.2+/-1.2	15	0.3+/-0.2	-34.0+/-3.0	-338.2+/-21.4	6.6+/-2.7	67.4+/-14.9	1260.6+/-27.1	-2.7
BindCraft 1	-78.9+/-6.7	8	0.5+/-0.3	-42.2+/-1.3	-276.3+/-32.0	8.0+/-2.6	105.6+/-39.5	1232.0+/-66.4	-2.3
BindCraft 2	-76.9+/-2.7	36	0.7+/-0.6	-18.4+/-7.0	-323.9+/-37.4	-2.4+/-2.5	86.2+/-13.9	925.9+/-83.5	-1.4
BindCraft 3	-78.5+/-1.8	52	4.8+/-0.2	-27.7+/-4.4	-301.4+/-27.8	-1.0+/-1.3	105.1+/-16.0	1127.6+/-15.2	-1.4
BindCraft 4	-94.8+/-4.9	6	0.8+/-0.5	-34.1+/-6.7	-350.7+/-42.0	0.5+/-3.2	89.4+/-27.9	1395.7+/-171.4	-2.3

### Contacts Analysis of the de Novo Designed Peptides Against LasR

**Fig. 3:**
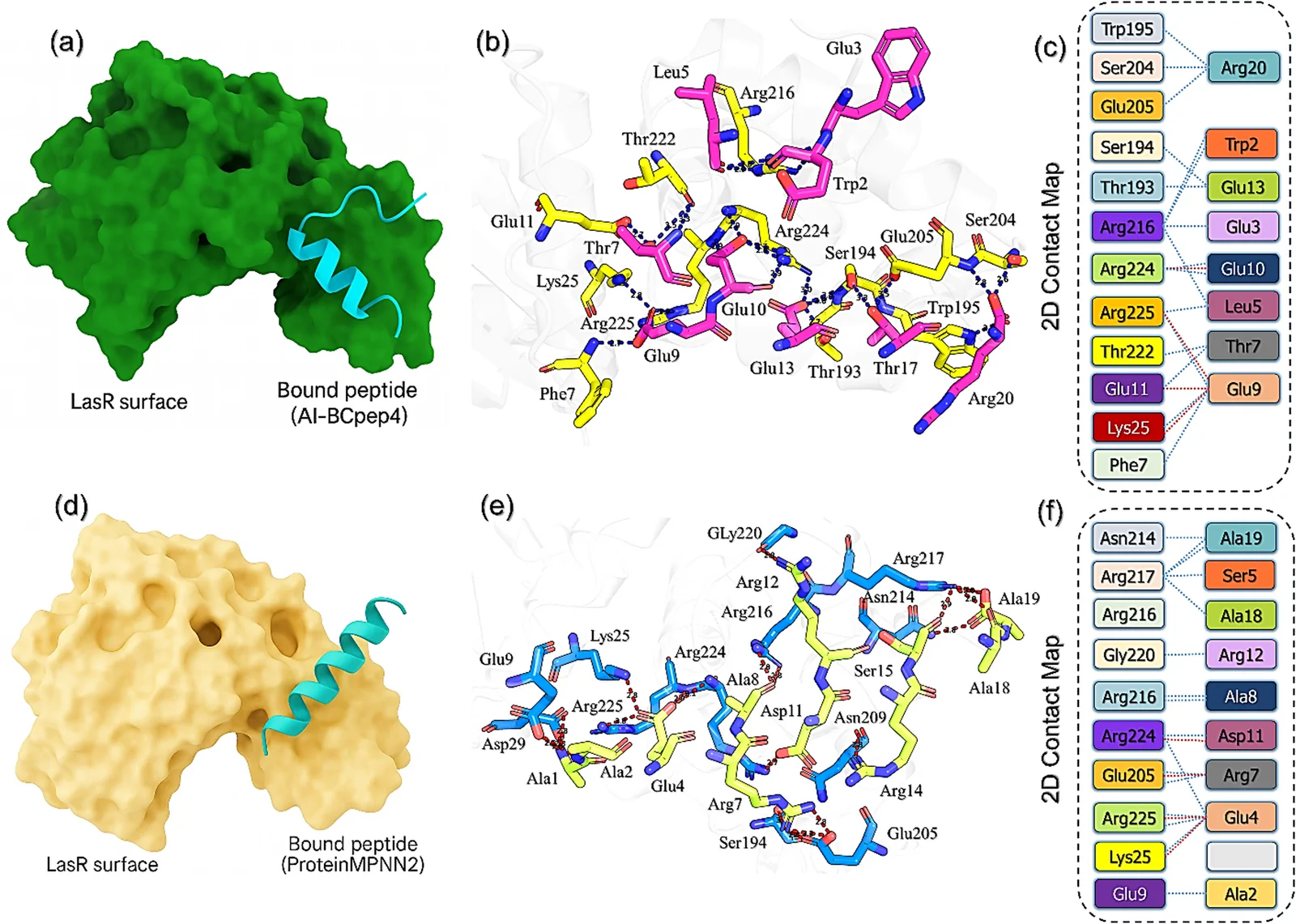
Contact map analysis between LasR with BindCraft4 and ProteinMPNN2 peptides. (**a**-**c**) shows the surface presentation of the Bindcraft4 peptide bound with the LasR interface, with the 3D interaction patterns and 2D contact map, while (**d**-**f**) shows the surface map, 3D interactions, and 2D contact map for the ProteinMPNN2-LasR complex. In the 2D contacts, the blue lines represent hydrogen bonds, while the red lines represent salt bridges

Docking analysis of the designed peptide library revealed substantial differences in energetic profiles and intermolecular interactions compared to the control peptide Aqs1C. Among the designed peptides, six showed lower HADDOCK scores than Aqs1C. We then thoroughly investigated the interaction patterns of the selected five peptides in complex with LasR to understand the binding differences compared to the control. For instance, the Bindcradft4-LasR complex reported a dense, most-occupied bonding network established by the essential hotspot residues. A total of 18 hydrogen bonds and 5 salt bridges were reported in the complex. At the N-terminal, Phe7-Glu9 (2.69 Å) and Glu11-Thr7 (2.68 Å) contacts were richly observed. Furthermore, the Glu205 region is indirectly involved via neighboring polar contacts, and the central clamp engages Arg216 with Trp2, Glu3, Leu5 (2.78–3.11 Å), and Arg224 with Glu10 and Glu13 (2.72–2.92 Å). This region is also accompanied by multiple salt bridges, including Arg224–Glu10 (2.72 Å), Arg225–Glu9 (2.76 Å), and between Lys25 and Glu9 (2.79 Å). The contact between Trp195 and Arg20 (2.84 Å) provides an additional polar anchor near the helix end. Altogether, BindCraft-4 combines rich Arg/Lys–Glu ionic pairs with additional HBs spanning Thr193, Ser194, Ser204, and Thr222, matching its strong electrostatic docking signature. The BindCraft4-LasR complex, as well as the 3D and 2D interactions contact maps, are shown in Fig. 3a and c.

On the other hand, the peptide, ProteinMPNN2, was ranked as the second most tightly bound and densely connected by the network of hydrogen bonds and salt bridges. This complex was predominantly occupied by a strongly electrostatically dominated network, as evidenced by the Glu205 residue establishing bidentate HBs and SBs with the peptide residue Arg7 (2.76–2.78 Å). Moreover, the residue Arg216 establishes two contacts with Ala8 (2.84 and 2.79 Å), while Arg224 engages Glu4 by forming a hydrogen bond (3.05 Å) and a salt bridge (2.71 Å). Arg225 also pairs with Glu4 (2.77–2.89 Å). Additionally, Gly220–Arg12 (2.76 Å) and Asn214–Ala19 (2.79 Å) are reported as essential contacts. These complex features include multiple reciprocal Arg–Glu salt bridges (Glu205–Arg7; Arg224/225–Glu4) along with Arg216–carbonyl clamps, consistent with the very strong electrostatic interactions observed in docking. The ProteinMPNN2-LasR complex, as well as the 3D and 2D interaction contact maps, are shown in Fig. 3d and f.

**Fig. 4:**
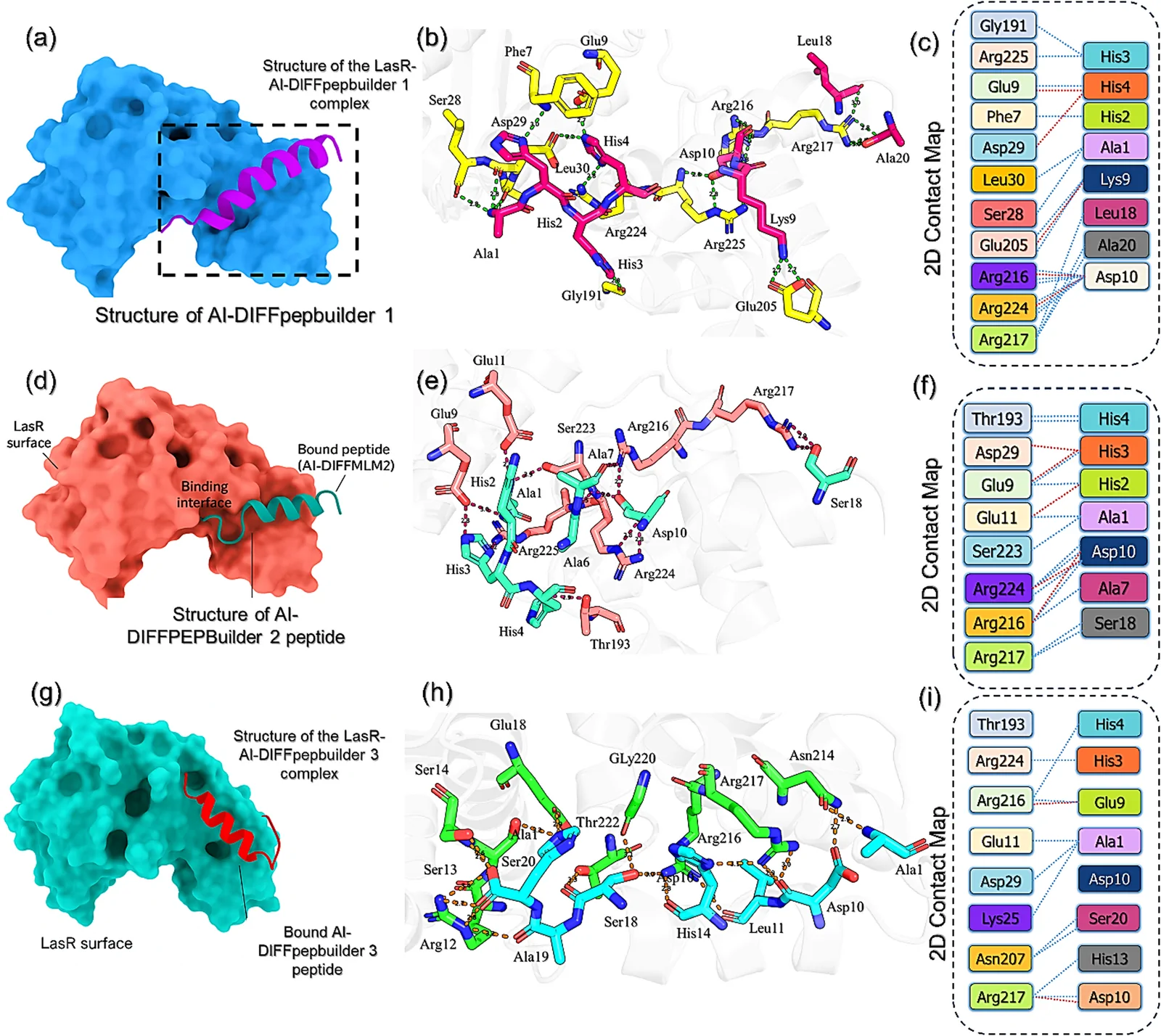
Contact map analysis between LasR and DiffPepBuilder1-3 peptides. (**a**-**c**) shows the surface presentation of the DiffPepBuilder1 peptide bound with the LasR interface, with the 3D interaction patterns and 2D contact map. (**d**-**f**) shows the surface map, 3D interactions, and 2D contact map for the DiffPepBuilder2-LasR complex. In contrast, (**g**-**i**) shows the surface map, 3D interactions, and 2D contact map for the DiffPepBuilder3-LasR complex. In the 2D contacts, the blue lines represent hydrogen bonds, while the red lines represent salt bridges

The complex formed by the peptide, DiffPepBuild1-LasR, exhibits a dense charge-balanced network interfacing across the canonical basic triad of LasR. Arg216 established multiple H-bonds to the peptide Asp10 (H-bonds ranged 2.80/2.75/2.85 Å), while Arg217 provided H-bonds to Leu18 and Ala20 (2.73–3.04 Å), anchoring the chain over the central interface. Arg224 engaged with Asp10 (H-bonds: 2.78/2.80/2.74 Å) and provided a salt bridge (Arg224-Asp10, 2.74 Å). For the acidic patch, Glu205 provided multiple H-bonds to Lys9 (two H-bonds, 2.66/2.73 Å) and a salt bridge (2.66 Å), anchoring the orientation of the N-terminus. Additional polar contacts were Gly191-His3 (2.78 Å), Glu9-His4 (H-bond and S.B, ~ 2.74 Å), and Leu30-Al1/Ser28 (2.73–2.83 Å). In total, DiffPepBuilder-1 demonstrated multivalent interactions with Arg216, Arg217, and Arg224, as well as with Glu205, resulting in strong electrostatic complementarity. The DiffPepBuilder1-LasR complex, along with the 3D and 2D interaction contact maps, is shown in Fig. 4a and c. In the complex DiffPepBuild2-LasR, Arg216 mediated the greatest contact with Ala7 and Asp10 (H-bonds 2.87/2.74 Å Shaw and S.B 2.74 Å), and Arg217 had dual H-bonds with Ser18 (2.87/2.99 Å). In the canonical triad, Arg224, coupled with Asp10 (H-bonds: 2.76/2.75 Å), and Ser223 made contact with Ala1 (2.90 Å). At the N-terminal acidic cluster, Glu9 and Glu11 provided multiple H-bonds to His2, His4, and Ala1 (H-bonds, 2.66–2.95 Å, and S.B). Glu11 interacted with His3 and His2 at 3.88 and 2.77 Å, respectively. The H-bond from Thr193-His4 (2.91 Å) provided additional stability to the helix-capping motif, which provided a thrust for a possible distinct orientation at the N-terminus. Within these orientations, this complex demonstrates distributed H-bonding across Arg216/217/224, involving concurrent engagement of the Glu–His ionic pairs, and fewer, but more secure, Lys–Glu clamps than in DiffPepBuilder1. The DiffPepBuilder2-LasR complex, along with the 3D and 2D interaction contact maps, is shown in Fig. 4d and f. Finally, the DiffPepBuild3-LasR complex also indicated a well-ordered network of a similar nature targeting Arg216/Arg217. Arg216 hydrogen-bonded to Glu9 and His4 (2.80/2.69 Å) and provided a salt-bridge with Glu9 (2.76 Å). Arg217, like DiffPepBuild1 and DiffPepBuild2, interacted with Asp10 (H-bond, 2.75 Å, and salt bridge, 2.75 Å), and also with His13 (2.78 Å). Additional network H-bonds and contacts included Asn207 with Ser20 (2.81/2.84 Å) and Lys25 with Ala1 (2.72 Å). Compared with DiffPepBuild1/2, DiffPepBuild3 demonstrated slightly tighter Arg216/217 anchoring and fewer Arg224 contacts, consistent with a more localized interface around a diffusible surface. The DiffPepBuilder3-LasR complex, along with the 3D and 2D interaction contact maps, is shown in Fig. 4g and i.

The remaining peptides, including DiffPepBuilder 2, BindCraft 1–3, ProteinMPNN 1, and EvoBind 2, display docking scores inferior to the control peptide, Aqs1C, in addition to forming fewer hydrogen bonds and salt bridges with the non-critical residues. These peptides typically did not consistently contact Arg224 and sometimes lost contact with Arg216 altogether. This explains the decrease in score and suggests that those designs destabilize the electrostatic clamp required for binding. Interestingly, peptides with scores higher than the control peptide showed more hydrogen bonds and stronger salt-bridge networks. For instance, expanded bonding networks, driven by both hydrogen bonds and salt bridges, have been observed to increase the binding strength of various biological molecules under different conditions [52, 53].

**Fig. 5:**
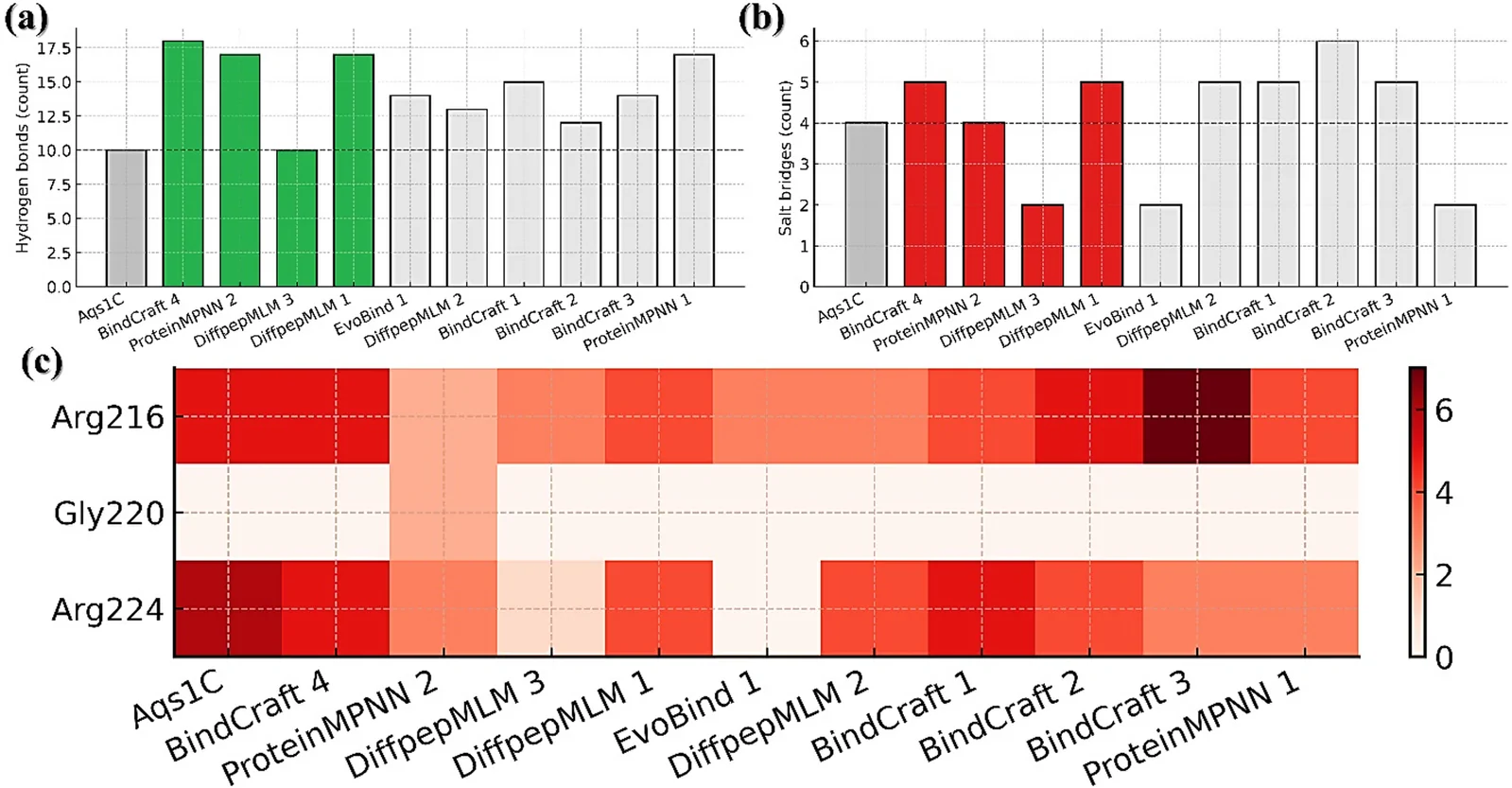
(**a**-**b**) represent the total hydrogen bonds and salt bridges count in each complex, while (**c**) shows the heatmap for key residues Arg216, Gly220, and Arg224 essential for inhibition

Overall, this reinforces that contacts with Arg216 and Arg224 remain the primary determinants of binding, while additional contacts with Gly220 can provide a layer of specificity. These findings align with the literature, which shows that disrupting the salt bridge at Arg224 eliminates peptide binding and that additional ion-pair networks are predictive of potency [12, 13]. Therefore, BindCraft 4, ProteinMPNN 2, DiffPepBuilder 3, and DiffPepBuilder 1 appear to be the best designs for experimental studies with EvoBind 1 as a secondary candidate. Peptides that do not reinforce Arg216/Arg224 are deprioritized. The hydrogen bonds and salt bridges involving key residues such as Arg216, Gly220, and Arg224 are shown in heatmaps for each complex in Fig. 5a and c. The detailed hydrogen-bonding interactions are provided in the **Supplementary Materials**.

### Binding Strength Re-Evaluation Through Dissociation Constant (Kd) Determination

To provide further insights into the binding strengths and differences between the control peptide and the novel *de novo*-designed peptides, we estimated interaction strength by predicting the dissociation constant (Kd). K_D_ is used to rank order and re-validate the strengths of biomolecular interactions [54]. It has been widely used in evaluations of antigen–antibody binding, protein–ligand interactions, and the estimation of affinity between large biological macromolecular complexes. The lower the Kd value, the stronger the interaction [52, 53]. The designed peptides reported significant improvement in the binding affinities compared to the native Aqs1C control (920 nM). Among the designed peptide candidates, DiffPepBuilder-2 had the best binding affinity, with a Kd of 28 nM, considered ultra-high, and a 30-fold increase relative to the native Aqs1C. On the other hand, DiffPepBuilder-1 determined a Kd of 56 nM. In comparison, BindCraft-4 reported a Kd of 77 nM and thus exhibits stronger binding affinities, with ~ 16- and 12-fold improvements, respectively, consistent with the higher structural fidelity observed due to its low pseudo-perplexity and stable interfacial contacts. Moreover, the peptide obtained from ProteinMPNN-2 reported a Kd of 140 nM, and DiffPepBuilder-3 determined 210 nM of Kd, thus exhibiting moderate-to-strong binding in contrast to the native Aqs1C control. These designed peptides showed significant improvements in binding strength with DiffPepBuilder, yielding the highest binding affinities, indicative of generative confidence and strong sequence-structure compatibility, as determined by the sequence programming. The affinity ranges produced by diffusion-based methods (DiffPepBuilder) yielded tighter complexes, ultimately due to the low-entropy geometrically optimized folds generated. At the same time, sequence optimization (ProteinMPNN) and reinforcement learning (BindCraft) also produced somewhat stronger binding affinities. In conclusion, all AI-derived peptides exhibited sub-micromolar Kd values, confirming their high-affinity binding and supporting the predictive value of the AI design pipelines. These observations provide quantitative evidence that independent AI-driven design peptides are structurally plausible, with binding energetics that are thermodynamically favorable and, in some cases, competitively higher than those of native peptide–protein binding complexes.

### Physicochemical Landscape of Designed Peptides

To thoroughly evaluate the physicochemical properties of *de novo* peptides designed by multimodal AI frameworks, we compared the physiochemical properties of these novel peptides with those of the native control peptide. The average length of peptides produced by these multimodal AI frameworks averaged 19.8 (residues) and was very similar to the control peptide, which had a length of 20 residues. Hence, the multimodal AI frameworks generated sequence-constrained peptides within the known natural size range for QSIs, which was an important aspect of peptide design. The molecular weight of *de novo* peptides (≈ 2.21 kDa) was slightly less than that of the control peptide (2.33 kDa), meaning a more compact peptide would be energetically favorable to synthesis and passive diffusion across bacterial membranes. The isoelectric point (pI) of the *de novo* peptides was moderate (pI ≈ 5.4) as opposed to the acidic control peptide (pI 4.4), which would lead to novel peptides that have a net-zero charge. Better solubility and optimized electrostatic complementarity to the LasR binding cavity could be achieved with a net-zero charge. The net charge of the *de novo* peptides (− 3.1) was similar to the control peptide (− 3.14), demonstrating that *de novo* peptides preserved the anionic behavior important for recognition of the receptor’s positively charged side chains Arg61 and Lys42 that have been previously reported to interact with the LasR–ligand structure. The GRAVY score of the *de novo* peptides (− 0.55) suggested a more balanced hydrophilic–hydrophobic backbone composition, leading to peptides that were slightly more stable in aqueous solutions than the native sequence, thereby improving solubility potential, while still having the potential to pack hydrophobically into the receptor pocket. Importantly, the *de novo* peptides had a higher aliphatic index than the control peptide (89.3 versus 78.5, respectively), revealing higher aliphatic side-chain content, such as Ala, Leu, Ile, and Val, increasing the thermostability of the *de novo* peptides, which is important for experimental validation of bioactivity in biological conditions. The instability index improved from 65.0 in the control to 41.8 in the designed sequence, thereby shifting it from “unstable” to “stable” according to in silico criteria. This improvement indicates the rigidity of the backbone and better propensity to resist spontaneous degradation compared to the native peptide, which aligns with the diffusion-based generation strategy’s focus on energetics realism. Furthermore, the fraction of the peptide that is classified as aromatic is reduced (0.02 vs. 0.10), and the corresponding improvement in extinction coefficients (≈ 1,400 M⁻¹ cm⁻¹ vs. 6,990 M⁻¹ cm⁻¹) suggests there were fewer bulky aromatic residues, which could ultimately improve folding efficiency and receptor accessibility. This aromatic content is an expected trait to achieve non-fluorescent, compact inhibitory peptides. Predictions of secondary structure indicated that the AI-designed peptides had a higher fraction of α-helices (0.58) than the control (0.45), while retaining stable proportions of β-sheet (0.22) and turn (0.14). This enrichment in α-helical content is advantageous for peptides that bind LasR structurally, as helices promote intermolecular hydrophobic interactions at the interface. The residue composition exhibited a balance between charged residues (≈ 45%) and hydrophobic residues (≈ 44%) while reducing polar residues (10% vs. 20%). This suggests an interface that will be more hydrophobic, allowing better docking to the hydrophobic core of the LasR protein. Thus, providing additional evidence that the AI-generated peptides were not random but rather physiochemically optimized, maintaining essential charge and polarity for LasR recognition while also improving overall structural stability and thermodynamic favorability. To quantify the propensity for the LasR–peptide complexes to aggregate in their folded conformations, we used Aggrescan4D (A4D) analysis. The control peptide (i.e., Aqs1C) displayed a lower tendency to aggregate with an average score of -0.4855 and a total score of -122.33, indicating a moderate hydrophobic cluster with some solubility. Among the peptides designed by AI, ProteinMPNN 2 had the greatest reduction (average − 2.3117), suggesting reduced surface hydrophobicity. This is a desirable characteristic that ensures solubility and folding fidelity. BindCraft 4 also improved with respect to the control (average − 1.6402), producing a balanced distribution of hydrophobic and hydrophilic amino acid residues and observable, stable tertiary compacted conformations. Conversely, the series of DiffPepBuilder peptides had a mixed result, matching the scoring of the Aqs1C with the aggregating potential, with a near-zero score (DiffPepBuilder1 (0.5802) & DiffPepBuilder 2 (0.4205)), suggesting a slightly positive tendency to aggregate risk, but also some folding instability. DiffPepBuilder peptide 3 (average − 0.5642) exhibited a drop below the total stability measure of the control. The results of the A4D were consistent with the physicochemical characterization we had identified previously for structure-based AI models for aggregation propensity (ProteinMPNN 2 > BindCraft 4 > Control > DiffPepBuilder 3 ≫ DiffPepBuilder1/2). The A4D results provide further evidence of reduced aggregate propensity, improved cooperativity during folding, and improved solubility for the designed peptides in the predicted conformational ensemble. The complete results are shown in **Supplementary Table **1.

### Structural-Dynamics Assessment of the LasR-peptide Complexes

#### Temporal Evolution and Stability Landscape of LasR–Bound Peptide ComplexesTem

**Fig. 6:**
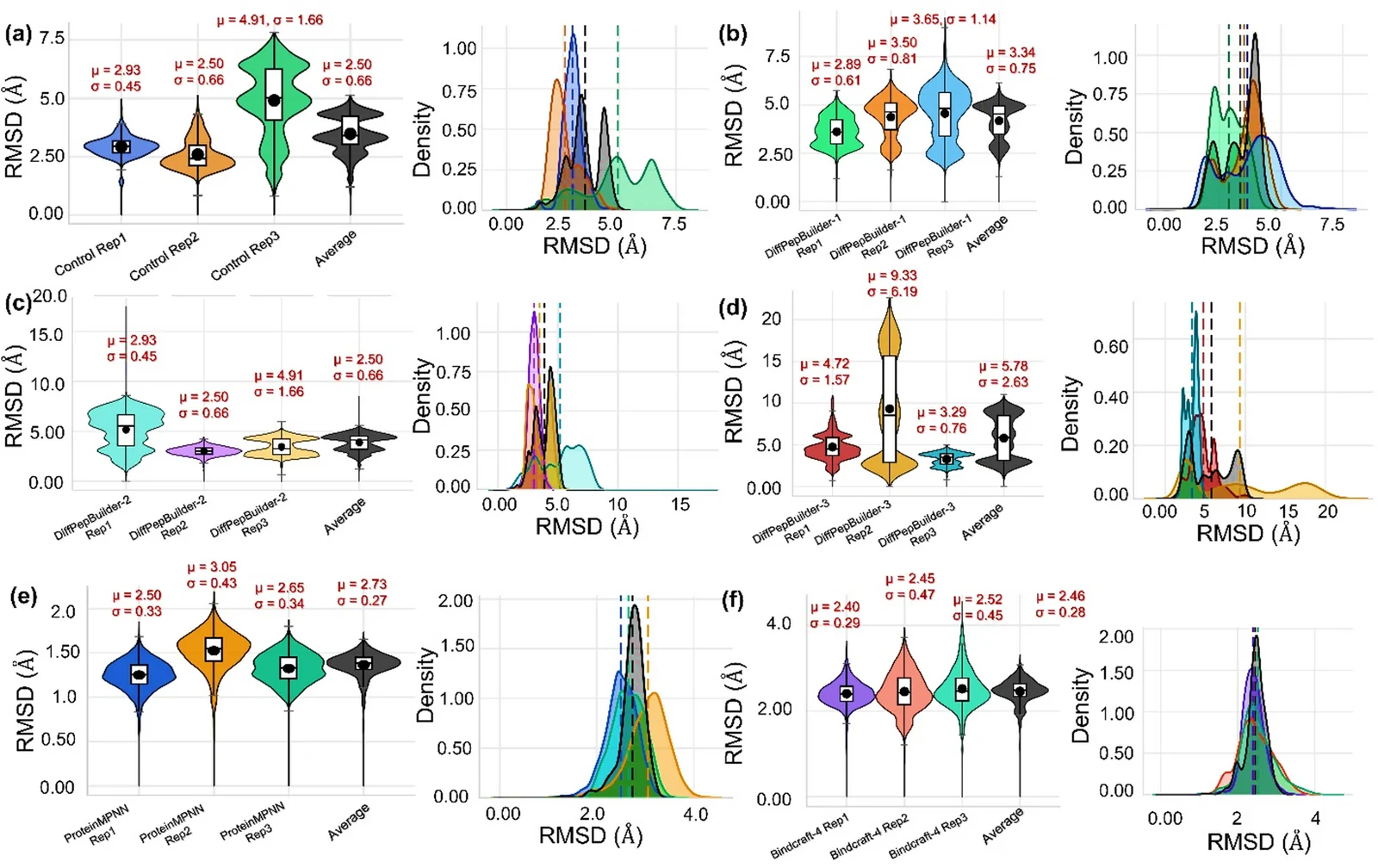
Dynamic stability analysis of the control and de novo designed peptides in complex with LasR. (**a**) shows the violin and density graphs for the Aqs1C-LasR (control) complex across all the replicas, (**b**-**d**) shows the violin and density graphs for the DiffPepBuilder1/2/3-LasR complexes across all the replicas, and (**e**) shows the violin and density graphs for the ProteinMPNN2-LasR complex across all the replicas. In contrast, (**f**) shows the violin and density graphs for the Bindcraft4-LasR complex across all the replicas. The mean (µ) and SD (σ) are also given on each leaf

An investigation of the structural dynamic features also provides conclusive information on the biological function or mechanism of an important cellular process. Using computational methods or algorithms to observe and investigate biomolecular dynamics provides a useful alternative to costly, time-consuming experimental procedures, especially for deciphering disease mechanisms and developing therapeutics at the atomic level. In this regard, RMSD is primarily used as a key metric to quantify structural stability and divergence from the initial conformation in different complexes, such as during folding and unfolding. The variations reported during the simulation determine the structural behavior of biological molecules relative to their conformations, with smaller deviations indicating greater structural stability and larger deviations indicating greater structural perturbation [52, 53, 55, 56]. Thus, herein we also used the same metric, i.e., RMSD from the Cα backbone, over the 400 ns (triplicate) trajectory of each complex. Across all systems, the MD trajectories reveal reproducible, yet design-specific RMSD behaviors that distinguish compact, well-equilibrated complexes from those that sample expanded or heterogeneous states. In the control peptide (Aqs1C), Fig. 6a, the three replicas cluster around distinct but moderate RMSD baselines with per-replica distributions of 2.93 ± 0.45 Å (rep1), 2.59 ± 0.66 Å (rep2), and 4.91 ± 1.52 Å (rep3), yielding an ensemble average of 3.47 ± 0.77 Å. The time series shows quasi-stationary plateaus with intermittent excursions, most prominently in rep3, consistent with the broader density tail and the higher mean for that replica. The Pearson matrix indicates only modest cross-replica agreement, reporting correlations of 0.50–0.62 between replica 1, replica 2, and replica 3, and ≥ 0.95 with the ensemble average. The distributions differ significantly by a Kruskal–Wallis test (H = 32106.150, *p* = 0.000e + 00). These baseline properties define a reference point against which the AI-designed sequences can be compared.

The peptides designed by the DiffPepBuilder algorithm in complex with LasR were also assessed for dynamic stability over a 400 ns simulation in triplicate. The Diffpepbuilder1 demonstrated a more similar dynamic stability behavior to the control but does not surpass the best performers. For the DiffPepBuilder1-LasR complex, the means across replicas were 2.89 ± 0.61 Å, 3.50 ± 0.81 Å, and 3.65 ± 1.14 Å, respectively, with an ensemble average of 3.34 ± 0.75 Å. The density profiles cluster around ~ 2–5 Å with modest expansion, and replica-to-replica Pearson coefficients are 0.63–0.67 with strong correlations to the average (0.87–1.00). These data indicate a compact ensemble, comparable to but slightly tighter than the control, due to the absence of a high-RMSD tail analogous to that in control replica 3. The RMSD graphs, presented as violin boxplots, and the density graphs for the DiffPepBuilder1-LasR complex are shown in Fig. 6b. On the other hand, the Diffpepbuilder2-LasR complex demonstrated heterogeneous behavior across the three replicas, with a higher-RMSD mode (replica 1, 5.18 ± 1.81 Å) and two tighter modes (replica 2, 3.01 ± 0.45 Å; replica 3, 3.45 ± 0.85 Å), yielding an ensemble average of 3.88 ± 0.84 Å. Moreover, the Pearson matrix makes the heterogeneity explicit (rep1–rep2 = 0.03; rep1–rep3 = 0.66; rep2–rep3 = 0.23; average correlations of 0.95, 0.21, and 0.82 with rep1–rep3), and the density plot shows a more extended, right-shifted tail driven by replica 1. This pattern indicates a design that intermittently explores expanded conformations, with only a subset of replicas occupying compact basins, as in the control. The RMSD graphs, presented as violin boxplots, and the density graphs for the DiffPepBuilder2-LasR complex are shown in Fig. 6c. We further demonstrated the dynamic behavior of Diffpepbuilder3-LasR, which showed comparatively worse behavior across all metrics. The replicas span 4.72 ± 1.57 Å, 9.33 ± 6.19 Å, and 3.29 ± 0.76 Å, with an ensemble average of 5.78 ± 2.63 Å. The kernel density reveals a heavy right-tail dominated by replica 2; the time series shows sustained excursions beyond ~ 10 Å; and the Kruskal–Wallis statistic confirms pronounced distributional differences (H = 14139.745, *p* = 0.000e + 00). The cross-replica Pearson structure (0.60–0.80 within, ≥ 0.86 to the average) indicates that the ensemble mean tracks a mixture of a compact sub-state and a persistent expanded state rather than a single stable basin. The RMSD graphs, presented as violin boxplots, and the density graphs for the DiffPepBuilder3-LasR complex are shown in Fig. 6c.

Finally, we also assessed the dynamic stability patterns by the two peptides, i.e., ProteinMPNN2 and BindCraft-4, in complex with LasR, which consistently outperform the control by combining low mean RMSD with lower deviations. In the case of ProteinMPNN2-LasR complex, the per-replica means RMSD for each complex was 2.50 ± 0.33 Å, 3.05 ± 0.43 Å, and 2.65 ± 0.34 Å, respectively, with an ensemble average of 2.73 ± 0.27 Å. The density curves are unimodal and narrowly peaked, and although cross-replica Pearson coefficients are only moderate (0.33–0.38), each replica correlates strongly with the ensemble average (0.66–0.83). The distributions are distinct by Kruskal–Wallis (H = 20722.421, *p* = 0.000e + 00), but the small σ values indicate that the differences reflect narrow, design-specific baselines rather than instability. On the other hand, BindCraft-4 demonstrates the lowest ensemble mean, i.e., 2.46 ± 0.28 Å with per-replica means of 2.40 ± 0.29 Å, 2.45 ± 0.47 Å, and 2.52 ± 0.45 Å. Despite relatively weak cross-replica correlations (0.19–0.26) that imply different dynamical pathways, each replica again correlates well with the average (0.56–0.75), and the distributions differ significantly (H = 843.007, *p* = 2.036e-182). Taken together, both ProteinMPNN2 and BindCraft-4 maintain stable conformational ensembles with small intra-replica variance and narrow kernel densities that remain centered between ~ 2.4 and 3.1 Å across time, indicating stable LasR–peptide assemblies that are more stable than the control ensemble (3.47 ± 0.77 Å). The RMSD graphs are shown as violin boxplots, with density graphs for the ProteinMPNN2 and Bindcraft4 peptides with the receptor LasR, in Fig. 6e and f.

Despite the differences in design, the temporal evolution confirms the distributional statistics, with ProteinMPNN2 and BindCraft-4 being the most stable constructs, exhibiting relatively smaller stability deviations (consistent with σ ≤ 0.47 Å across replicas). In contrast, DiffPepBuilder-3 exhibits periodic high-RMSD behavior, whereas DiffPepBuilder-2 transitions between compact and expanded conformations across replicas. DiffPepBuilder-1 and the control occupy intermediate ground; their trajectories are predominantly stationary with occasional modest excursions, but neither achieves the combination of low mean and low dispersion observed for ProteinMPNN2 and BindCraft-4. Statistically, within-peptide variability (replica-to-replica) is smallest for ProteinMPNN2 and BindCraft-4, which maintain tight µ and σ values across replicas; it is moderate for DiffPepBuilder-1 and the control, and highest for DiffPepBuilder-2 and DiffPepBuilder-3, which exhibit pronounced dispersion and notable mismatch among replicas. These patterns suggest stronger and more persistent interaction networks for the ProteinMPNN2–LasR and BindCraft-4–LasR complexes, whereas DiffPepBuilder-3 and DiffPepBuilder-2 display evidence of sampling partially dissociated or more solvent-exposed states. Mechanistically, the more dynamically stable peptides, i.e., ProteinMPNN2 and BindCraft-4, likely maintain a contiguous hydrophobic core and facilitate recurrent interfacial contacts, thereby limiting global motions, consistent with low σ values and unimodal density distributions. In contrast, the comparatively unstable variants permit large-scale breathing motions and transitions to higher-energy states. This behavior is reflected in their right-skewed distributions, shifting baselines across replicas, and weak inter-replicate correlations. Overall, the MD data provide compelling evidence that ProteinMPNN2 and BindCraft-4 are the most promising designs to advance, whereas DiffPepBuilder-3 should be deprioritized, and DiffPepBuilder-2 should be redesigned to suppress the expanded basin captured by its high-RMSD replica.

#### Conformational Folding and Expansion of the Peptide-LasR complexes

**Fig. 7:**
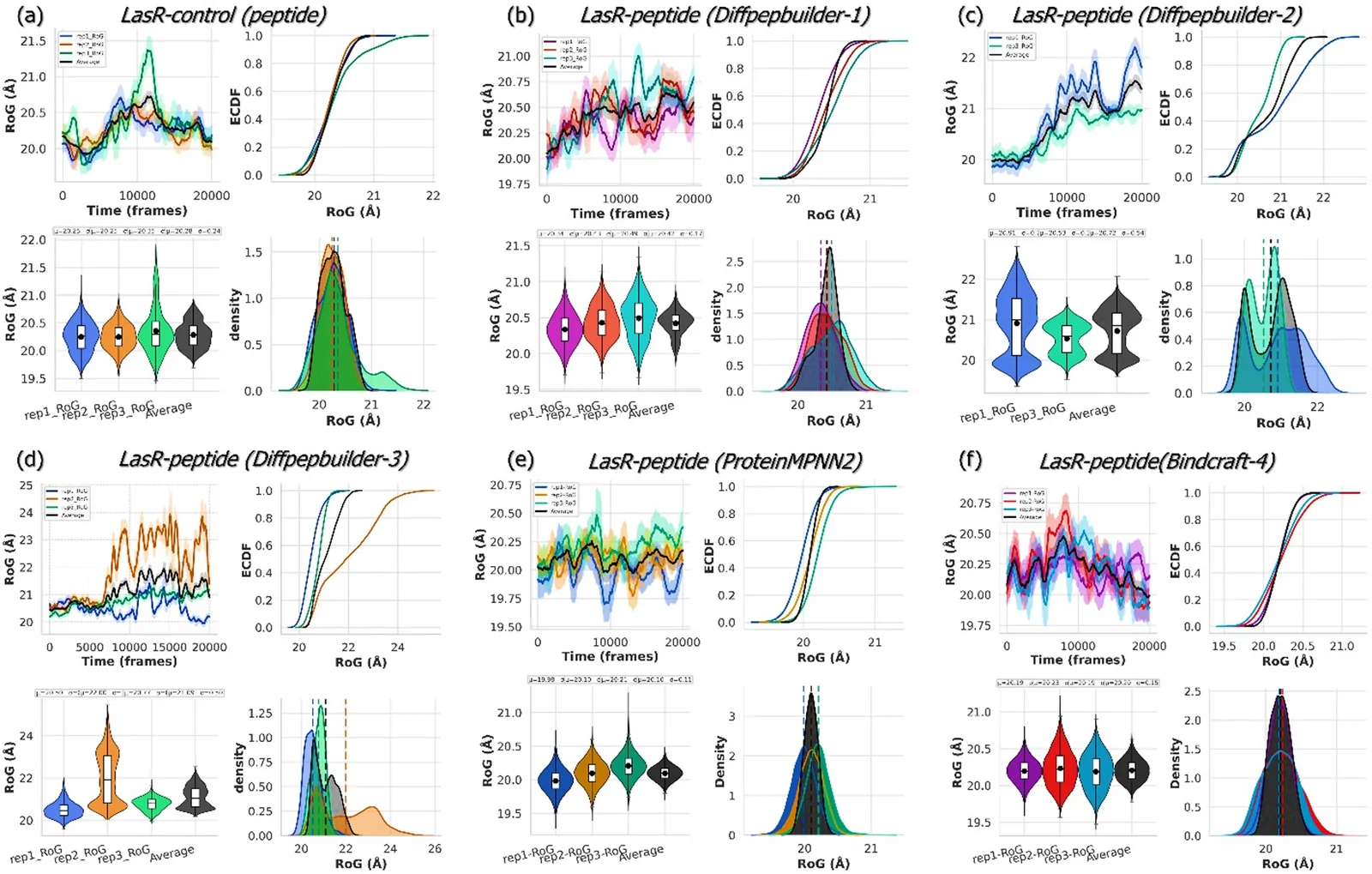
Radius of gyration (RoG) profiles of LasR–peptide complexes. Panels **a–f** shows the control and designed peptides (Diffpep-1 to 3, ProteinMPNN2, Bindcraft-4). Each line represents one replica, with shaded bands showing mean ± SD. Compact, overlapping traces indicate convergence, whereas broader variations reflect increased conformational flexibility

The radius of gyration (RoG) analysis provided in Fig. 7a and f comprehensively describe the structural compactness of the LasR receptor in complex with control and designed peptides over extended MD simulations. Across all systems, RoG were evaluated across three independent replicas, complemented by cumulative distribution functions (ECDFs), violin plots, and kernel density plots to assess ensemble convergence and structural heterogeneity. In the LasR-control peptide complex **(**Fig. 7a**)**, all three replicas exhibited highly consistent temporal profiles, converging rapidly within the first 5000 frames and stabilizing near 20.3 Å. The narrow-shaded envelopes around each trajectory indicate minimal frame-to-frame variability, confirming structural equilibrium of the native receptor–peptide assembly.

The violin plot was sharply unimodal, and the ECDF curve increase steeply, revealing a homogeneous and compact ensemble. This system, therefore, represents a stable reference baseline with negligible tertiary expansion during equilibration. The LasR–Diffpepbuilder-1 complex **(**Fig. 7b**)** displayed a slightly higher mean RoG of approximately 20.6 Å, with mild oscillations persisting throughout the simulation. The shaded bands across replicas remained moderately narrow, suggesting that the structural deviations were reversible and likely correspond to peptide-induced motions rather than unfolding. The ECDF remained steep, although right-shifted relative to control, consistent with a minor relaxation of the LasR fold upon peptide association. For LasR–Diffpepbuilder-2 **(**Fig. 7c**)**, RoG initially tracked closely with control for the first half of the trajectory before a gradual rise beyond 21.2 Å, accompanied by increasing replica divergence. The broader violin distribution and slightly flattened ECDF slope reflect increased configurational sampling and a transition toward a more open conformational state. These features imply moderate destabilization of the receptor core or enhanced local fluctuations in the ligand-binding domain. The LasR–Diffpepbuilder-3 complex **(**Fig. 7d**)** demonstrated the most pronounced deviation among all designs. Replica trajectories fluctuated between 20 Å and 23.5 Å without achieving clear convergence, and the average trace exhibited sustained drift throughout the simulation. The wide, bimodal density profile signifies the coexistence of compact and expanded states, while the shallow ECDF slope confirms structural heterogeneity. Such persistent variability suggests incomplete equilibration and partial disassembly of inter-domain contacts, likely arising from suboptimal peptide anchoring or excessive conformational flexibility introduced by the Diffpep sequence. By contrast, the LasR–ProteinMPNN2 complex **(**Fig. 7e**)** converged strongly across all replicas, maintaining RoG values between 20.2 and 20.5 Å with small, consistent standard deviations. The ensemble mean was nearly flat beyond 8000 frames, and both violin and density plots were unimodal and narrow. The ECDF showed a steep rise centered at ~ 20.3 Å, similar to that of the control complex. These findings indicate that ProteinMPNN2 stabilizes the LasR tertiary structure, maintaining near-native compactness and minimized entropic fluctuations, attributes consistent with favorable, well-packed binding conformations predicted for machine-learning-derived peptides. Finally, the LasR–Bindcraft-4 complex **(**Fig. 7f**)** exhibited moderate oscillations within 20.4–21.0 Å, yet the replicas remained well overlapped and converged. The density distribution was unimodal but broader than the control, suggesting a slightly more flexible but still compact state. The ECDF slope lay between those of ProteinMPNN2 and Diffpep-1, implying controlled mobility rather than destabilization. The RoG trends across all systems follow the order of increasing compactness and stability: Control, ProteinMPNN2, Bindcraft-4, Diffpep-1, Diffpep-2, Diffpep-3. The control and ProteinMPNN2 complexes exhibited the lowest mean RoG and narrowest distributions, confirming robust tertiary packing and equilibrium convergence.

In contrast, the DiffPepBuilder series, particularly Diffpep-3, showed pronounced structural expansion and inter-replica divergence, reflecting higher conformational entropy and weaker intramolecular cohesion. These results suggest that among the designed peptides, ProteinMPNN2 most effectively preserves LasR’s native structural compactness, consistent with a stable, well-defined binding interface. In contrast, the DiffPepBuilder constructs tend to induce dynamic loosening and partial unfolding of the receptor architecture.

#### Residual Flexibility Assessment

**Fig. 8:**
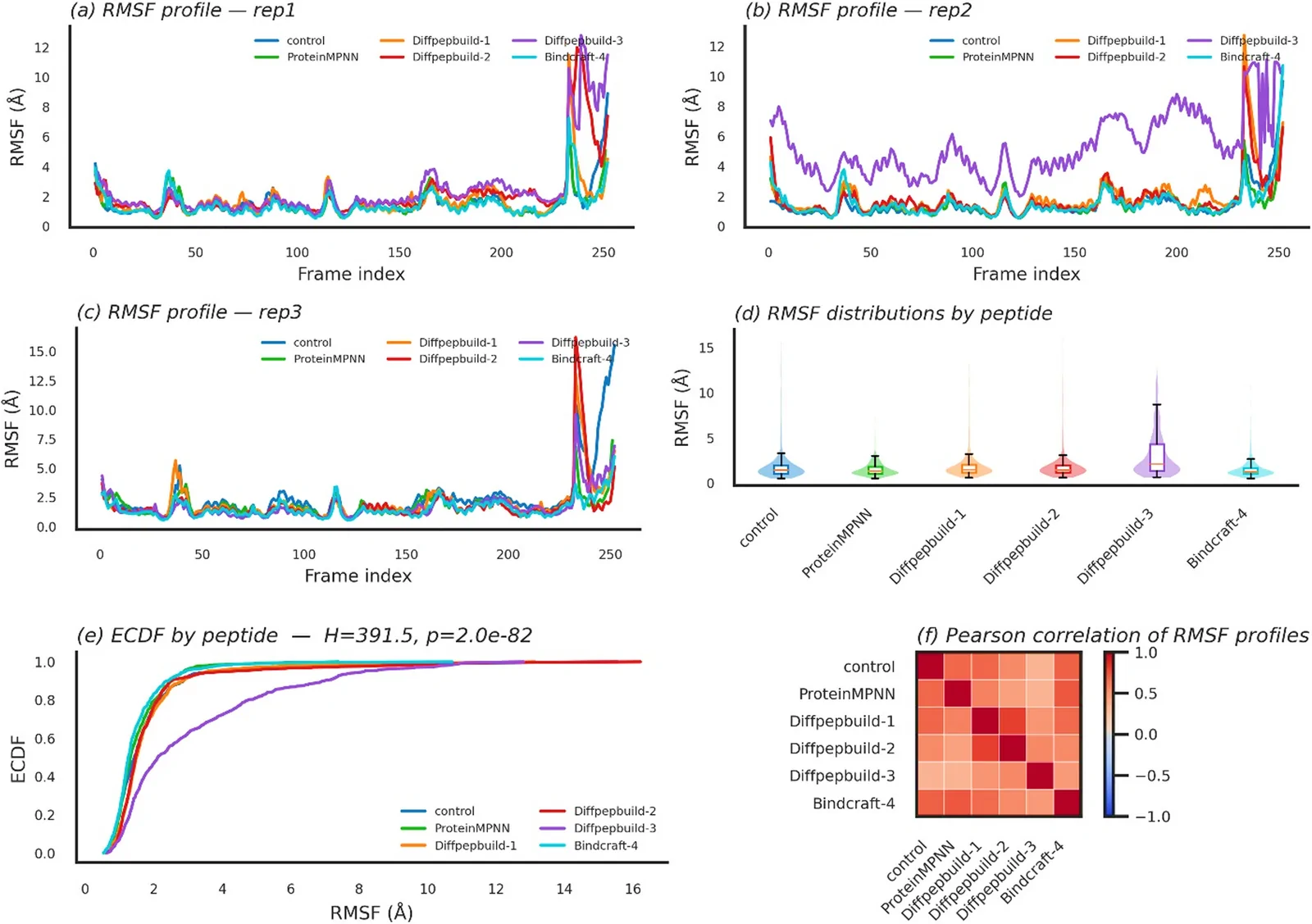
Residual flexibility analysis of the control and de novo designed peptides in complex with LasR. (**a**) shows the RMSF graphs for all the complexes across replica 1, (**b**) shows the RMSF graphs for all the complexes across replica 2, (**c**) shows the RMSF graphs for all the complexes across replica 3, (**d**) shows the RMSF distribution graphs for all the peptides, and (**e**) shows the ECDF graph for all the complexes. In contrast, (**f**) shows the Pearson correlation of RMSF profiles

The RMSF analyses performed for the three independent replicas **(**Fig. 8a and c**)** show reproducible residue-level fluctuations that define the conformational landscapes of the control peptide (Aqs1C) and AI-designed peptides (DiffpepMLM-1-3, ProteinMPNN-2, and BindCraft-4). Each trajectory converged after equilibration, and the mean RMSF amplitudes were stable across the three replicas, confirming both trajectory reproducibility and convergence quality. Residue positions 35–55 and 82–95 consistently manifested as highly flexible regions corresponding to loop-like regions at the LasR binding interface that are believed to undergo breathing motions during complex stabilization. All peptides showed these local peaks, but the amplitudes varied significantly across the design algorithms.

The control Aqs1C displayed relatively low per-residue fluctuations (~ 1.4–1.9 Å) throughout the chain, indicative of a reasonable flexibility typical of an evolved binder that can accommodate flexibility to its binding conformations without jeopardizing the integrity of the binding interface. The machine-learning-derived DiffpepMLM-1 and DiffpepMLM-2, on the other hand, showed less fluctuation over the same residues (mean 1.2 ± 0.3 Å), implying that these designs limited fluctuations in the core area, leading to greater packing density. The second ProteinMPNN construct, ProteinMPNN-2, had an almost identical RMSF profile as the control Aqs1C, particularly illustrated in the 41–46 and 88–93 residues, suggesting that a changed one-dimensional sequence did not inhibit the binding construct’s ability to stabilize the binding topology while still enabling the overall dynamic envelope to remain with the same profile. Differently, DiffpepMLM-3 and BindCraft-4 exhibited larger spikes of residue deviation greater than roughly 3 Å at residues 50–53 (site Phe50) and 86–90 (site Phe3), indicating a less favorable intermediary motion between the β-turn either at the specific residues or segmental flexibility of the entire β-turn structure (please see Models 3 and 4). Localized fluctuations, not observed in the next most stable protein constructs, may suggest the same limitations in packing density and saturation of the binding interface that are not experienced in DiffpepMLM-2 and ProteinMPNN-2, respectively. RMSF distributions averaged across the four replicas **(**Fig. 8d**)** supported the previous observations, showing that the trend behaviors were statistically significant. Specifically, while DiffpepMLM-2 and ProteinMPNN-2 violin plots showed narrow, unimodal distributions, DiffpepMLM-3 and BindCraft-4 showed broader tail segments at higher RMSF fluctuations. Additionally, the Kruskal-Wallis test (H = 14 139.745, *p* < 10 − 4) indicated that the overall variability observed among the four peptides is highly significant and that the null hypothesis of identical populations is rejected. Finally, the non-parametric evidence supports the idea that the disparate rigidity of each protein complex arises from likely true redesign-dependent differences in binding, rather than random noise or differences across replicas. The ECDF curves **(**Fig. 8e**)** provide an integrated visualization of cumulative backbone stability. The steep, left-shifted curves of DiffpepMLM-2 and ProteinMPNN-2 indicate that > 80% of their residues fluctuate within 1.5 Å, a hallmark of well-folded, conformationally restrained complexes. The flatter profiles of DiffpepMLM-3 and BindCraft-4, in contrast, reflect larger fractions of residues with RMSF > 2.5 Å, aligning with the localized peaks in their residue-wise maps. The correspondence between the ECDF slope and the violin-plot spread confirms that the mechanical rigidity inferred from local RMSF maxima extends to the global backbone stability. The Pearson correlation heatmap **(**Fig. 8f**)** underscores cross-peptide similarity in fluctuation patterns. DiffpepMLM-2 and ProteinMPNN-2 correlate strongly with each other (*r* = 0.98) and with the control (*r* = 0.93), reinforcing that both designs recapitulate the control’s intrinsic dynamic signature. DiffpepMLM-3, by contrast, shows a markedly reduced correlation (*r* = 0.68) with all other systems, signifying a divergent flexibility regime likely driven by altered loop dynamics. BindCraft-4’s intermediate correlation (*r* ≈ 0.79) suggests partial mimicry of the native conformational pattern but insufficient rigidity at specific hinge points. Residue-specific hotspot mapping (from averaged RMSF across replicas) further refines these insights.

In Aqs1C, peaks centered at Gly45, Ser48, and Lys89 coincide with solvent-exposed loops predicted to tolerate motion without destabilizing the core. DiffpepMLM-2 and ProteinMPNN-2 suppress these peaks to < 1.5 Å, implying more robust intra-helical packing or reinforced hydrogen-bond networks in the designed sequences. In contrast, DiffpepMLM-3 exhibits exaggerated motion at Asp51–Thr54, aligning with the interface periphery, potentially disrupting key hydrophobic contacts. BindCraft-4 shows heightened mobility at Leu87–Val90, consistent with weaker side-chain burial at the C-terminal binding motif. These spatially localized fluctuations are mechanistically consistent with transient breathing of the LasR–peptide interface: successful designs confine motion to peripheral loops, whereas unstable ones allow motion to propagate toward the core interface. Collectively, these results establish a hierarchy of dynamic stability: ProteinMPNN-2 ≈ DiffpepMLM-2 > Aqs1C > DiffpepMLM-1 > BindCraft-4 > DiffpepMLM-3, reflecting how distinct AI-driven design frameworks shape the complex’s energetic landscape. The RMSF profiles reveal broadly conserved fluctuation patterns across all complexes, indicating overall structural stability of the protein–peptide assemblies. Notably, increased fluctuations observed near residue ~ 250 correspond to the C-terminal regions of the peptides, which differ in sequence composition among the control and designed peptides. These variations reflect intrinsic peptide-specific flexibility rather than destabilization of the binding interface. Despite these localized differences, the core regions of the peptides display comparable RMSF behavior, consistent with preserved binding modes and stable complex formation. The convergence of per-residue RMSF peaks, pooled distributions, ECDF slopes, and inter-peptide correlations validates that deep-learning-based design can indeed reproduce (and occasionally surpass) the native dynamic phenotype when structural constraints are implicitly learned. Simultaneously, the statistical divergence among variants highlights that generative models remain sensitive to local sequence inaccuracies that amplify motion in critical interface loops. Thus, multi-replica MD combined with rigorous fluctuation analysis provides a quantitative dynamic filter for distinguishing functionally viable peptide designs from nominal docking predictions.

### Thermodynamic Binding Free-Energy Analysis Across Simulation Timescales

The early-stage MMGBSA binding free energy profiles extracted from the first 10 ns of the MD trajectories provide an initial measure of binding stability and interaction energetics for the LasR–peptide complexes before full equilibration. MM-GBSA is a widely used, computationally efficient endpoint method whose relative binding free energies often correlate well with experimental trends when applied consistently to a given system. Multiple benchmarking and challenge studies have shown that, despite method-dependent absolute values, MM-GBSA can reach experimentally meaningful accuracy for ranking ligands and reproducing mutation-induced affinity changes, making it a dependable filter before costly assays [57–59]. Therefore, we used this approach to estimate the strength of our design peptides against LasR.

The control peptide (Aqs1C) established a benchmark mean total binding free energy (ΔG total) of − 33.6 ± 7.3 kcal/mol. Its interaction energy was driven by substantial vdw (− 53 kcal/mol) and electrostatic (− 360 kcal/mol) attractions, partially offset by polar solvation penalties (~ 388 kcal/mol). The moderate standard deviation suggests early stabilization after minor conformational relaxation. This energetic balance demonstrates a native-like complex that maintains strong intermolecular complementarity during the initial phase of MD sampling.

Among the AI-generated designs, ProteinMPNN 2 displayed an almost identical early binding profile (ΔG_total_ = − 33.4 ± 8.5 kcal/mol), indicating that its redesigned interface preserved the overall electrostatic and hydrophobic characteristics of the control while possibly achieving a slightly smoother solvation response. The comparable magnitude and variance imply that the ProteinMPNN sequence can attain a similarly equilibrated binding mode even within the short 10 ns window, suggesting favorable folding kinetics and stable complex formation. BindCraft 4, in contrast, exhibited the most favorable energetic signature during this initial sampling period, with an average ΔG_total_ of − 46.4 ± 2.6 kcal/mol, approximately 13 kcal/mol stronger than the control. This enhancement originates from deeper vdw minima and modest electrostatic contributions (− 442 kcal/mol) that are not excessively penalized by desolvation (ΔG_solv_ ≈ 454 kcal/mol). The low standard deviation across replicas reflects a consistently compact binding geometry. These features together indicate rapid stabilization of a hydrophobically optimized complex and suggest that BindCraft 4 likely forms a pre-organized interface even before full conformational equilibration, a hallmark of strong and persistent binding.

In contrast, the DiffPepBuilder series showed progressively weaker stabilization. DiffPepBuilder 1 and 2 exhibited moderate affinities of − 21.3 ± 7.8 kcal/mol and − 21.8 ± 5.4 kcal/mol, respectively, characterized by diminished electrostatic attraction and larger polar solvation costs. Their trajectories likely sample more flexible or partially disengaged conformations during this early period, consistent with incomplete adaptation of the peptide termini or insufficient burial of charged residues. DiffPepBuilder 3 showed the weakest binding (ΔG_total_ = − 17.5 ± 3.9 kcal/mol), with electrostatics reduced to roughly one-third of the control’s magnitude and minimal compensatory van der Waals stabilization. Despite this lower affinity, the narrow spread among its three replicas indicates reproducible sampling of a relatively shallow binding mode rather than random fluctuations, confirming that its weaker interaction is a real energetic property rather than noise. Overall, these early MMGBSA estimates reveal that within the first 10 ns, the binding strength hierarchy is already well defined: BindCraft 4, ProteinMPNN 2, Control, DiffPepBuilder 1, DiffPepBuilder 2, DiffPepBuilder 3. This ranking highlights the strong initial stabilization of the BindCraft design, the near-native robustness of the ProteinMPNN-generated peptide, and the comparatively loose, energetically penalized associations formed by the DiffPepBuilder variants. The trends suggest that BindCraft 4 is the most promising candidate for sustained binding over longer trajectories. In contrast, DiffPepBuilder sequences will likely require further optimization to achieve control-level stability in subsequent simulation stages. The binding free energy for each replica, along with the mean values, is given in Table 2.

**Table 2:** Binding free energy results for each complex across all the replicas, and the mean binding free energy values with standard deviation (SD) calculated are given. The results are from the first 10 Ns of the simulation trajectory for each replica

Complex	Replica	VDW	EEL	EGB	ESURF	ΔG_gas_	ΔG_solv_	ΔG_total_
Control (Aqs1C)	1	−55.92	−375.11	405.32	−7.71	−431.05	397.61	**−33.44**
	2	−42.92	−321.41	354.30	−6.19	−364.33	348.10	**−16.23**
	3	−63.25	−383.02	403.84	−8.76	−446.26	395.08	**−51.18**
	**Mean ± SD**	**−53.36 ± 10.23**	**−359.85 ± 31.64**	**387.82 ± 26.65**	**−7.55 ± 1.30**	**−413.88 ± 42.15**	**380.26 ± 28.25**	**−33.62 ± 7.28**
ProteinMPNN 2	1	−47.41	−355.92	377.20	−7.86	−403.34	369.34	**−34.01**
	2	−54.96	−315.55	349.79	−9.12	−370.51	340.67	**−29.84**
	3	−58.28	−323.09	354.73	−9.55	−381.38	345.17	**−36.21**
	**Mean ± SD**	**−56.88 ± 5.48**	**−331.52 ± 21.17**	**360.57 ± 15.02**	**−8.85 ± 0.91**	**−385.08 ± 17.37**	**351.06 ± 14.97**	**−33.35 ± 8.50**
BindCraft 4	1	−58.10	−441.90	462.80	−9.07	−500.00	453.73	**−46.27**
	2	−59.32	−435.75	460.12	−8.89	−495.07	451.23	**−43.84**
	3	−57.44	−448.36	466.01	−9.21	−505.80	456.80	**−49.00**
	**Mean ± SD**	**−58.29 ± 0.94**	**−441.94 ± 6.30**	**462.98 ± 3.00**	**−9.06 ± 0.16**	**−500.29 ± 5.36**	**453.92 ± 2.72**	**−46.37 ± 2.59**
DiffPepBuilder 1	1	−58.57	−293.86	340.80	−7.89	−352.44	332.91	**−19.53**
	2	−58.62	−329.31	374.64	−8.10	−387.94	366.53	**−21.40**
	3	−56.61	−363.09	404.62	−7.79	−419.71	396.82	**−22.89**
	**Mean ± SD**	**−57.93 ± 1.10**	**−328.75 ± 34.61**	**373.35 ± 31.92**	**−7.93 ± 0.15**	**−388.38 ± 33.65**	**365.42 ± 32.04**	**−21.27 ± 7.79**
DiffPepBuilder 2	1	−56.24	−394.39	427.59	−8.56	−450.64	419.03	**−31.60**
	2	−29.20	−232.10	251.30	−4.00	−261.32	247.30	**−14.02**
	3	−50.30	−259.67	297.08	−6.81	−309.98	290.28	**−19.71**
	**Mean ± SD**	**−45.25 ± 13.92**	**−295.39 ± 84.69**	**325.33 ± 88.69**	**−6.46 ± 2.33**	**−340.64 ± 96.41**	**318.87 ± 86.03**	**−21.78 ± 5.35**
DiffPepBuilder 3	1	−43.62	−149.57	177.48	−6.23	−193.19	171.25	**−21.95**
	2	−42.59	−96.94	130.73	−5.69	−139.53	125.04	**−14.50**
	3	−40.28	−130.58	160.39	−5.47	−170.87	154.92	**−15.95**
	**Mean ± SD**	**−42.16 ± 1.68**	**−125.70 ± 26.89**	**156.20 ± 24.76**	**−5.80 ± 0.40**	**−167.86 ± 26.90**	**150.40 ± 23.40**	**−17.47 ± 3.90**

All complexes maintained thermodynamic stability, indicating convergence toward equilibrium binding. BindCraft 4 remained the strongest binder (− 46.0 ± 5.3 kcal/mol), displaying robust van der Waals and electrostatic components (ΔG_gas_ ≈ − 489 kcal/mol) partially offset by solvation (ΔG_solv_ ≈ + 469 kcal/mol). This suggests persistent hydrophobic packing and salt-bridge stabilization even in late dynamics. Control (Aqs1C) retained moderate binding (− 22.9 ± 12.7 kcal/mol) with higher fluctuation, confirming its weaker interface stability compared to engineered peptides. DiffPepBuilder 1 and 2 showed consistent but variable affinity ( ≈ − 21 kcal/mol) with some improvement in Replica 2 of DiffPepBuilder 1, implying enhanced conformational adaptation. ProteinMPNN 2 remained stable and reproducible (− 17.8 ± 2.6 kcal/mol), while DiffPepBuilder 3 equilibrated at − 16.8 ± 12.8 kcal/mol, suggesting partial stabilization after early flexibility. The binding free energy for each replica, along with the mean values, is given in Table 3. Throughout both simulation intervals, BindCraft 4 exhibited the most robust and energetically preferred binding behavior, as evidenced by all replicates and time intervals showing strongly negative ΔG_total_ values. The persistent energetic advantage BindCraft 4 experienced was due to complementary contributions from strong van der Waals packing and stabilizing electrostatic interactions that remained balanced throughout the simulation, even in the presence of solvent relaxation effects late in the intervals. However, the control complex (Aqs1C) showed a further decline in binding affinity as it progressed through the final 10 ns simulation window, indicative of typical conformational relaxation and partial loss of interfacial contacts. Both DiffPepBuilder variants (1 and 2) maintained moderate and consistent binding affinity through both time intervals, suggesting that the receptor interface interactions were pliable but stable enough to demonstrate continued binding and could provide a structural scaffold for optimization of side-chain composition or charge distribution. ProteinMPNN 2 and DiffPepBuilder 3 showed a noticeable decline in binding stability over time, losing some electrostatic contributions and gaining contributions from solvation penalties and solvent effects due to greater solvent exposure of key residues anchoring to the protein interface. Together, these findings highlight unique dynamic responses to the introduction of AI-engineered peptides: BindCraft 4 forms a tightly packed, energetically converged complex; DiffPepBuilder variants exhibit stable yet pliable binding; and ProteinMPNN 2 is sensitive to desolvation at the interfacial region, suggesting that AI-engineered peptides stabilize peptide-receptor binding via distinct energetic mechanisms.

**Table 3:** Binding free energy results for each complex across all the replicas, and the mean binding free energy values with standard deviation (SD) calculated are given. The results are from the last 10 Ns of the simulation trajectory for each replica

Complex	Replica	VDW	EEL	EGB	ESURF	ΔGgas	ΔGsolv	ΔGtotal
Control (Aqs1C)	1	−31.43	−461.52	458.35	−5.38	−492.96	452.97	**−39.98**
	2	−23.95	−254.70	263.18	−3.54	−278.66	259.64	**−19.02**
	3	−28.99	−171.93	194.77	−3.69	−200.93	191.08	**−9.85**
	**Mean ± SD**	−28.13 ± 3.77	−296.05 ± 147.4	305.43 ± 136.5	−4.20 ± 0.99	−324.18 ± 146.8	301.23 ± 131.9	**−22.95 ± 12.7**
ProteinMPNN 2	1	−48.64	−206.42	247.46	−6.49	−255.06	240.97	**−14.10**
	2	−42.87	−182.54	212.89	−5.73	−225.43	207.16	**−18.26**
	3	−49.83	−162.74	200.53	−6.92	−212.58	193.61	**−18.97**
	**Mean ± SD**	−47.11 ± 3.73	−183.90 ± 21.9	220.29 ± 24.2	−6.38 ± 0.62	−231.02 ± 21.3	213.91 ± 24.2	**−17.78 ± 2.59**
BindCraft 4	1	−53.02	−431.38	472.07	−7.83	−484.40	464.24	**−40.16**
	2	−57.88	−440.63	485.91	−8.59	−498.51	477.32	**−47.22**
	3	−54.91	−429.32	474.18	−7.84	−484.23	466.33	**−50.60**
	**Mean ± SD**	−55.27 ± 2.45	−433.78 ± 5.84	477.39 ± 6.90	−8.09 ± 0.43	−489.05 ± 8.05	469.30 ± 6.77	**−46.00 ± 5.25**
DiffPepBuilder 1	1	−52.59	−261.86	306.96	−6.96	−314.45	299.99	**−14.46**
	2	−52.49	−313.50	336.83	−7.61	−366.00	329.22	**−36.78**
	3	−38.97	−181.40	212.20	−5.05	−220.38	207.15	**−13.23**
	**Mean ± SD**	−48.02 ± 8.14	−252.25 ± 66.5	285.33 ± 65.0	−6.54 ± 1.35	−300.28 ± 73.8	278.79 ± 65.4	**−21.49 ± 13.4**
DiffPepBuilder 2	1	−16.89	−220.67	225.14	−2.86	−237.57	222.28	**−15.29**
	2	−31.86	−245.63	270.47	−4.57	−277.49	265.91	**−11.59**
	3	−42.55	−252.92	266.49	−5.89	−295.48	260.60	**−34.88**
	**Mean ± SD**	−30.43 ± 12.88	−239.74 ± 16.7	254.03 ± 23.8	−4.44 ± 1.52	−270.18 ± 29.4	249.60 ± 23.0	**−20.59 ± 12.1**
DiffPepBuilder 3	1	−27.78	−232.76	236.71	−3.81	−260.55	232.91	**−27.65**
	2	−16.43	−195.64	210.43	−3.25	−212.07	207.18	**−2.48**
	3	−23.12	−220.50	240.23	−4.14	−243.63	236.09	**−20.37**
	**Mean ± SD**	−22.44 ± 5.67	−216.30 ± 18.7	229.12 ± 16.2	−3.73 ± 0.46	−238.08 ± 24.4	225.39 ± 14.7	**−16.83 ± 12.8**

### Essential Dynamics and Trajectories Motion

**Fig. 9:**
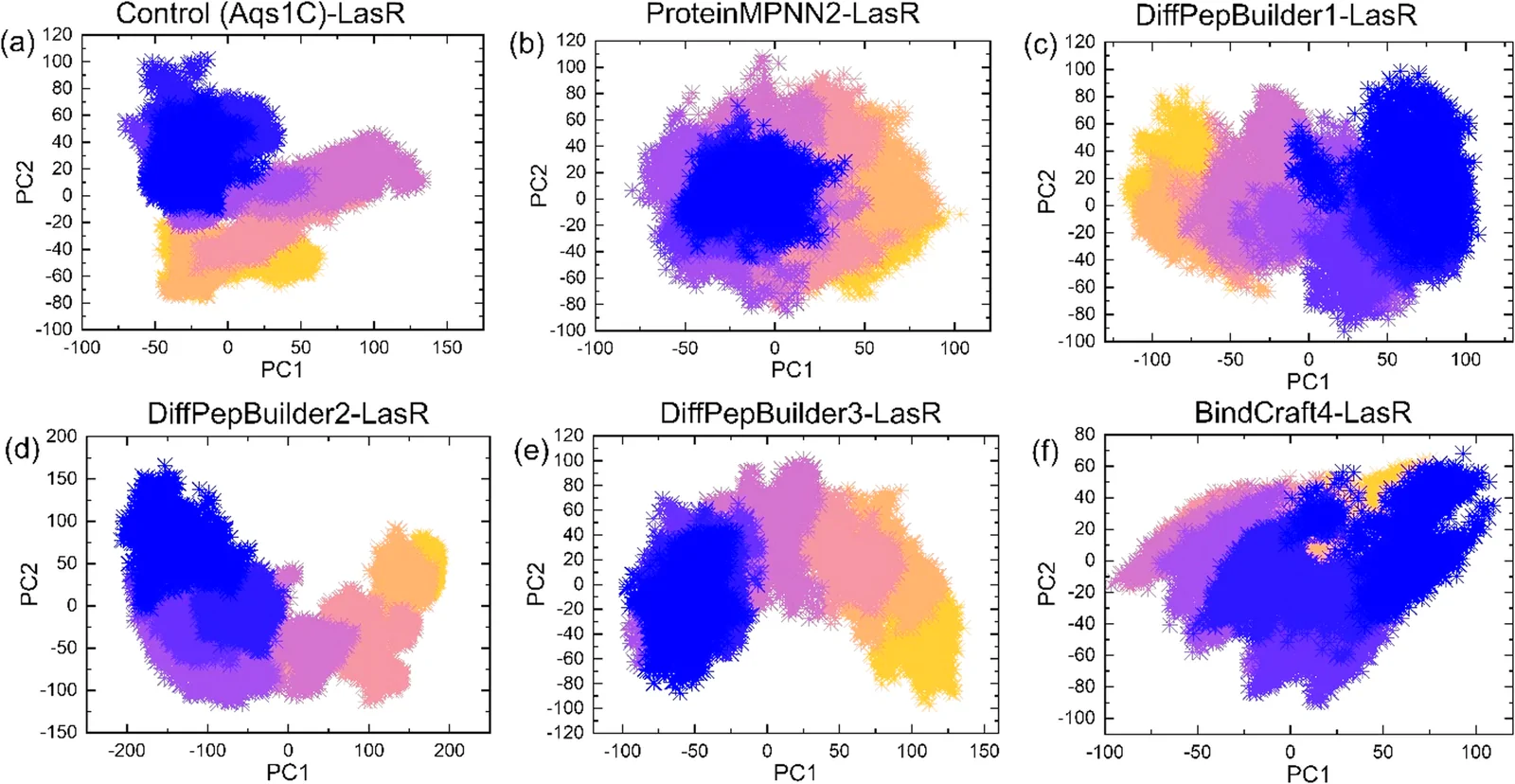
Principal component analysis of the control and designed peptides in complex with LasR. (**a**) shows the trajectories distribution for the control (Aqs1C) in complex with LasR, (**b**) shows the trajectories motion for ProteinMPNN2-LasR complex, (**c**-**e**) shows the distribution of trajectories for DiffPepBuilder1/2/3 in complex with LasR. In contrast, (**f**) shows the trajectory motion for the BindCraft-designed peptide in complex with LasR

To study the collective atomic motions and the major conformational modes of the LasR–peptide complexes in the simulation trajectories, Principal Component Analysis (PCA) was utilized. The projection of the first two principal components (PC1 and PC2) clearly shows a variation in conformational sampling behaviors among the systems. The control complex (Aqs1C–LasR) showed significant dispersion across the PC1–PC2 plane, with numerous overlapping clusters reflecting high conformational diversity and transitions among metastable states. The large spread in conformations reflects only moderate structural stability, as observed previously from the RMSD and MMGBSA analyses. The ProteinMPNN2–LasR and DiffPepBuilder1–LasR complexes exhibited relatively minimal PCA dispersion and clustering, suggesting that these systems sampled more stable structural ensembles while globally restricting motion, albeit with moderate energy stability. The DiffPepBuilder2 and DiffPepBuilder3 systems displayed more extensive trajectory sampling. Still, they had relatively separable clusters, suggesting greater conformational flexibility and loop dynamics, enabling potentially possible transient interactions that are not persistent at the receptor interface. The BindCraft4–LasR complex appears to have the most constrained PCA cluster, suggesting severe constraint in conformational subspace and strong internal stabilization reflecting even dominant binding stability and compactness among all systems. The PCA graphs for all the complexes are given in Fig. 9a and f.

**Fig. 10:**
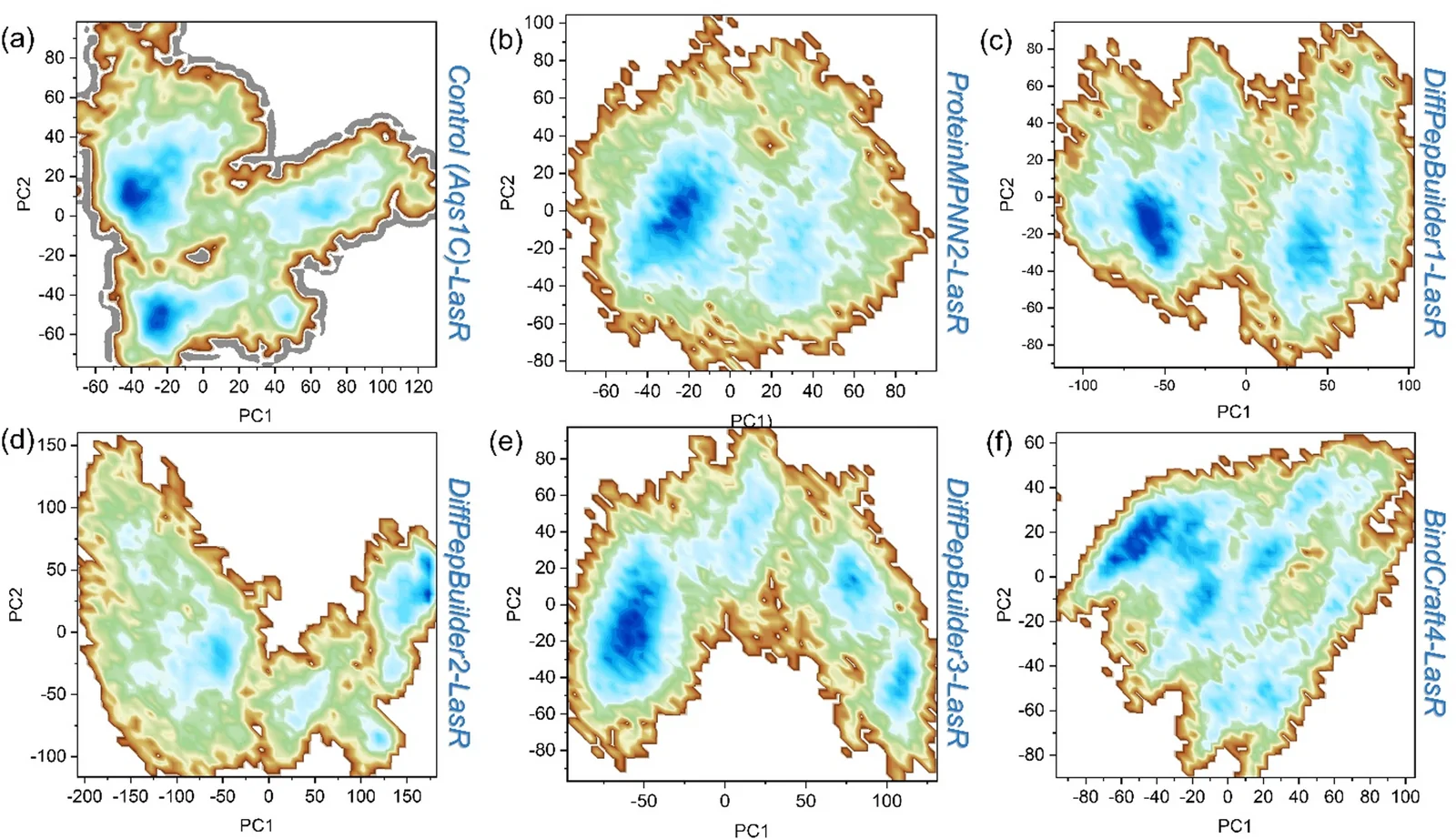
Free energy analysis of the control and designed peptides in complex with LasR. (**a**) shows the FEL graph for the control (Aqs1C) in complex with LasR, (**b**) shows the FEL graph for ProteinMPNN2-LasR complex, (**c**-**e**) shows the FEL graphs for DiffPepBuilder1/2/3 in complex with LasR, while (**f**) shows the FEL graph for the BindCraft-designed peptide in complex with LasR

The two-dimensional free energy landscapes (FELs) derived from the first two principal components further elucidated the conformational thermodynamics of each complex. The control system (Aqs1C–LasR), despite having multiple shallow basins connected by low-energy barriers, indicative of relatively continuous transitions between loosely packed conformations, highlights its dynamic and less stable complexity. ProteinMPNN2–LasR and DiffPepBuilder1–LasR were observed to have two moderate-deep basins, indicating a small selection of energetically viable substates separated by low barriers, characteristic of partially stable conformational equilibria. The FELs of DiffPepBuilder2 and DiffPepBuilder3 were more rugged, with several small energy wells across a larger PC1–PC2 space, indicative of increased conformational entropy and backbone flexibility. BindCraft4–LasR was characterized by a single deep and narrow energy minimum, indicating a strongly stabilized low-energy conformation with minimal configurational fluctuations. The highly converged basin for BindCraft4 confirms that it sustains a compact, energetically optimized structural ensemble that closely matches its superior ΔG_total_ and minimal RMSF fluctuations, further justifying BindCraft4 as the most dynamically stable and thermodynamically favorable peptide complex. The FEL graphs for all the complexes are given in Fig. 10a and f.

## Conclusions

This work establishes a unified AI–MD framework for peptide-based inhibitor discovery against LasR. AI-generated peptides demonstrated tunable dynamic stability, where BindCraft 4 consistently outperformed others in docking affinity and conformational convergence. The DiffPepBuilder 1–2 complexes exhibited promising flexibility for scaffold refinement, while ProteinMPNN 2 and DiffPepBuilder 3 showed partial destabilization after relaxation. The control peptide underwent natural unfolding and energy loss. These findings collectively affirm that AI-designed peptides can surpass natural templates by achieving enhanced binding stability, optimized interface packing, and favorable solvation energetics, providing a blueprint for next-generation QSIs. The results provided here were generated by AI models and physics-based simulations evaluation and rank the designed peptides, which need experimental validation for the potential clinical candidates designing.

## Supplementary Information

Below is the link to the electronic supplementary material.

Supplementary File 1 (DOCX 63.9 KB)

## Data Availability

No datasets were generated or analysed during the current study.
