# Supplementary material for: Bridging Generative AI and Diffusion Models With Molecular Simulation to Design Anti-Quorum-Sensing De novo Peptides Targeting LasR of Pseudomonas aeruginosa
Source: Probiotics Antimicrob Proteins. 2026 Mar 14;18(6):7605–30. doi: 10.1007/s12602-026-10956-5 (PMC13518548; doi:10.1007/s12602-026-10956-5)
Supplement: Supplementary file 1 [file 12602_2026_10956_MOESM1_ESM.docx]

**Supplementary Materials**

**Bridging Generative AI and Diffusion models with Molecular Simulation to Design Anti-Quorum-Sensing *De Novo* Peptides Targeting LasR** **of** ***Pseudomonas aeruginosa***

Abbas Khan^1^, Mona A. Sawali^1^, Anwar Mohammad^2^, Fakhrul Hasssan^3,^ Fahad M. Alshabrmi^4^, Eid A. Alatawi^5^, Sergio Crovella^6^, Dong-Qing Wei^7^, Abdelali Agouni^1*^

1. Department of Pharmaceutical Sciences, College of Pharmacy, QU Health, Qatar University, P.O. Box 2713, Doha, Qatar. [Abbas.khan@qu.edu.qa](mailto:Abbas.khan@qu.edu.qa); [aagouni@qu.edu.qa](mailto:aagouni@qu.edu.qa)
2. Precision Health Analysis Unit, Translational Research, Dasman Diabetes Institute, Dasman, Kuwait. [Anwar.mohammad@dasmaninstitute.org](mailto:Anwar.mohammad@dasmaninstitute.org)
3. Faculty of Rehabilitation and Allied Health Sciences, Riphah International University, Islamabad, Pakistan. [fakhr.hassan@riphah.edu.pk](mailto:fakhr.hassan@riphah.edu.pk)
4. Department of Medical Laboratories, College of Applied Medical Sciences, Qassim University, 51452 Buraydah, Saudi Arabia.
5. Department of Medical Laboratory Technology, Faculty of Applied Medical Sciences, University of Tabuk, Tabuk 71491, Saudi Arabia
6. Department of Biomedical Sciences, College of Health Sciences, QU Health, Qatar University, P.O. Box 2713, Doha, Qatar. [sgrovella@qu.edu.qa](mailto:sgrovella@qu.edu.qa)
7. Department of Bioinformatics and Biological Statistics, College of Life Sciences and Biotechnology, Shanghai Jiao Tong University, Shanghai, P.R. China. [dqwei@sjtu.edu.cn](mailto:dqwei@sjtu.edu.cn)

***Correspondence should be sent to:** Prof. Abdelali Agouni, Department of Pharmaceutical Sciences, College of Pharmacy, Qatar University, P.O. Box 2713, Doha, Qatar. **Tel:** +974 4403 5610; **Email:** [aagouni@qu.edu.qa](mailto:aagouni@qu.edu.qa)

**Materials and Methods**

**Data Normalization and Averaging Details**

To ensure reproducibility and comparability across all replicas and peptide systems, RMSD and RMSF values were subjected to rigorous statistical normalization and ensemble averaging. All quantitative data processing was carried out in Python 3.12 using NumPy, pandas, and SciPy libraries, following best practices for ensemble-based MD analysis.

**Per-Replica Normalization**

For each replica i of a given complex, residue-wise RMSF values were first aligned to a common reference frame after removal of translational and rotational degrees of freedom. Each trajectory was then centered and mass-weighted before fluctuation analysis. The raw RMSF per residue rⱼⁱ was calculated as:

$$RMSFⱼⁱ = \surd(1/T \Sigmaₜ₌₁ᵀ (rⱼ(t) - \langle rⱼ\rangle)^{2})$$

where rⱼ(t) is the position of atom j at time t, ⟨rⱼ⟩ is the time-averaged position, and T is the total number of frames post-equilibration. To avoid bias from differences in absolute displacement scales, each trajectory’s RMSF distribution was normalized by its median absolute deviation (MAD):

$$RMSFₙₒᵣₘⁱ = (RMSFⁱ - ṜMSFⁱ) / MADⁱ$$

This normalization ensured scale invariance across replicas and complexes, facilitating inter-system comparisons.

**Ensemble Averaging and Error Estimation**

Normalized RMSF data from all three replicas were combined residue-wise to compute the ensemble mean and variance:

$$RMSF̄ⱼ = (1/N) \Sigmaᵢ₌₁ᴺ RMSFⱼⁱ$$

$$\sigmaⱼ = \surd((1/(N-1)) \Sigmaᵢ₌₁ᴺ (RMSFⱼⁱ - RMSF̄ⱼ)^{2})$$

where, N = 3 (number of replicas). The reported RMSF values in all visualizations thus represent the mean ± standard deviation across independent trajectories, providing a quantitative measure of both the local flexibility and the reproducibility of residue fluctuations. For global comparison across peptides, per-residue means were integrated to yield the average RMSF for each complex:

$$\langle RMSF\rangle₍cₒₘₚₗₑₓ₎ = (1/M) \Sigmaⱼ₌₁ᴹ RMSF̄ⱼ$$

where M is the total number of residues considered in the complex. Standard errors of the mean (SEM) were computed as σⱼ / √N, and 95% confidence intervals were obtained assuming a Student’s t-distribution.

**Inter-Replica Correlation and Statistical Consistency**

To validate reproducibility between replicas, pairwise Pearson correlation coefficients (r) were computed from the residue-wise RMSF vectors:

$$rₐb = \Sigmaⱼ ((RMSFⱼᵃ - RMSF̄ᵃ)(RMSFⱼᵇ - RMSF̄ᵇ)) / \surd(\Sigmaⱼ (RMSFⱼᵃ - RMSF̄ᵃ)^{2} \Sigmaⱼ (RMSFⱼᵇ - RMSF̄ᵇ)^{2})$$

Correlation values greater than 0.9 confirmed excellent reproducibility, while cross-peptide correlation matrices were used to assess the similarity of fluctuation topologies among AI-designed peptides and the control. The strong intra-peptide correlations (0.86–0.98) validated consistent RMSF behavior across replicas, while inter-peptide correlations ranging from 0.68 to 0.93 reflected sequence- and topology-dependent dynamic divergence.

**Non-Parametric Distributional Testing**

All RMSF values were pooled across replicas for each peptide, and Kruskal–Wallis nonparametric tests were applied to determine whether the distributions originated from the same population. The resulting test statistic H = 14,139.745 (p < 10⁻⁴) indicated statistically significant variability in atomic fluctuation amplitudes between peptides. This approach avoided assumptions of normality inherent to parametric tests, thereby ensuring robust statistical discrimination of backbone flexibility patterns.

**Visualization and Error Representation**

All analytical plots were produced using Matplotlib (v3.9) and Seaborn (v0.13). Line plots represent mean RMSF per residue with shaded envelopes corresponding to ±1 standard deviation. Violin plots depict the density and spread of RMSF distributions across peptides. ECDF curves quantify cumulative backbone rigidity, highlighting the fraction of residues within each fluctuation threshold. Correlation heatmaps summarize reproducibility and design similarity. All figures were rendered at 300 dpi with white backgrounds and publication-safe color palettes.

**Table S1:** The table shows all the designed peptides using ProteinMPNN with their scores and sequence IDs.

| Index | PPS | Scores | Designed Peptide Sequences |
| --- | --- | --- | --- |
| 1 | 1.543 | 0.187 | AAGAAVREAADRARSEAAA |
| 2 | 1.568 | 0.187 | AAGEAVRAAADRARSEAAA |
| 3 | 1.583 | 0.125 | AAGEAVRRARDEAASRRAA |
| 4 | 1.583 | 0.25 | AAGAAVRDAADRAASLAAA |
| 5 | 1.588 | 0.187 | AAGEAVRAARDRARSEAAA |
| 6 | 1.588 | 0.125 | AAGEAVRRTRDEAASRAAA |
| 7 | 1.589 | 0.125 | AAGEAVRRARDEAASRRAA |
| 8 | 1.598 | 0.187 | AAGAAVREARDRARSEAAA |
| 9 | 1.601 | 0.187 | GAGAAVRAARDAARSRAAA |
| 10 | 1.614 | 0.187 | AAGDAVRAAADRAASLAAA |
| 11 | 1.618 | 0.187 | AAGAAVREAADRARSIAAA |
| 12 | 1.631 | 0.062 | AAGEAVRRTADEARSRRAA |
| 13 | 1.632 | 0.125 | AAGDRVRLTADRARSEAAA |
| 14 | 1.634 | 0.062 | AEGEEVRRRADEEASRRAA |
| 15 | 1.635 | 0.125 | AAGEAVRRTRDTAASLAAA |
| 16 | 1.635 | 0.125 | AEGEAARRTRDAARSRAAA |
| 17 | 1.638 | 0.125 | AAGEAVRATRDRARSEAAA |
| 18 | 1.647 | 0.125 | AAGEAVRLTRDRAASEAAA |
| 19 | 1.649 | 0.125 | AAGDAVRLAADRARSERAA |
| 20 | 1.652 | 0.125 | GEGERVRRTRDERESRERA |
| 21 | 1.662 | 0.125 | GAGERVRRTADERRSRAAA |
| 22 | 1.662 | 0.062 | ERGEEVRRRRDEERSRREA |
| 23 | 1.671 | 0.125 | AAGEAVRRAADARRSRLAA |
| 24 | 1.672 | 0.062 | AAGELVRRTADERRSREAA |
| 25 | 1.672 | 0.062 | AAGERVRLTADRRRSEEAA |
| 26 | 1.676 | 0.187 | AEGEAARLARDRAESERRA |
| 27 | 1.676 | 0.125 | AEGEAVRLAADRRRSEEAA |
| 28 | 1.677 | 0.125 | AEGEAVRRRRDLARSRAAA |
| 29 | 1.678 | 0 | EEGERVRLERDRRRSEEER |
| 30 | 1.682 | 0.125 | AAGTAVRLTRDTAASVAAA |
| 31 | 1.683 | 0.125 | AAGDRVRLTRDRAASEAAA |
| 32 | 1.684 | 0.062 | AEGERVRRERDARLSREAA |
| 33 | 1.685 | 0.062 | EEGERVRLERDRRRSVEAA |
| 34 | 1.686 | 0.125 | AAGEAVRRAADSRRSRLAA |
| 35 | 1.691 | 0.125 | AAGEAVRLSADRARSLAAA |
| 36 | 1.693 | 0.125 | GEGEAVRLARDRRLSEEAA |
| 37 | 1.694 | 0.062 | ERGARVRETADRRRSELAA |
| 38 | 1.699 | 0.062 | AAGEQVRLTADRRRSELAA |
| 39 | 1.701 | 0.125 | AEGDLVRLTADRARSEAAA |
| 40 | 1.701 | 0.062 | GAGERVRLTRDLAASRRAA |
| 41 | 1.703 | 0.125 | AAGEAVRLAADRARSLRAA |
| 42 | 1.71 | 0.062 | EEGERVRLEADRRRSEEAA |
| 43 | 1.712 | 0.062 | EEGERVRLEADRRRSIEAA |
| 44 | 1.714 | 0.125 | AAGEQVRLTADTARSIAAA |
| 45 | 1.714 | 0.062 | EEGERVRLTRDRERSEREA |
| 46 | 1.714 | 0.062 | AAGELVRRTADARRSRLAA |
| 47 | 1.714 | 0.125 | ARGERERLTRDRRESEERA |
| 48 | 1.716 | 0.125 | ASGELVRRTADEAASRAAA |
| 49 | 1.719 | 0.125 | GLGERARLTADRARSLAAA |
| 50 | 1.721 | 0.125 | ARGERVRLEADRRRSEAAA |
| 51 | 1.722 | 0.062 | DAGERVRLTRDRALSERRA |
| 52 | 1.724 | 0.125 | AEGELVRRTRDRAESERAA |
| 53 | 1.726 | 0.125 | AAGEAVRLTADRRRSIAAA |
| 54 | 1.726 | 0.062 | ARGELVRRTRDERRSREAA |
| 55 | 1.73 | 0.062 | ARGELERLTRDRRLSELAA |
| 56 | 1.731 | 0.125 | ALGEAARLAADRRRSLLAA |
| 57 | 1.732 | 0.062 | ERGTRVRETRDRARSEREA |
| 58 | 1.741 | 0.125 | ARGELARLTRDRARSEAAA |
| 59 | 1.749 | 0.062 | SAGERVRLEADRRRSELAA |
| 60 | 1.75 | 0.062 | LAGELVRRTADERRSREAA |
| 61 | 1.751 | 0.125 | EEGELARLTRDRRESEERA |
| 62 | 1.752 | 0.125 | AEGELARLTEDRARSLAAA |
| 63 | 1.753 | 0.062 | AEGERVRLTADRARSLRAA |
| 64 | 1.754 | 0 | AEGERVRLTEDRRRSEEAR |
| 65 | 1.756 | 0.125 | AAGTAVRAARDSRRSREAA |
| 66 | 1.756 | 0.062 | EEGELVRLTRDRRLSEERA |
| 67 | 1.757 | 0.062 | AEGELVRLTADRRRSLEAA |
| 68 | 1.76 | 0.125 | ARGSRVRETRDRRESIERA |
| 69 | 1.761 | 0.062 | AEGEEVRLTRDRRRSLEAA |
| 70 | 1.761 | 0.062 | AEGELVRRTRDSRRSREAA |
| 71 | 1.761 | 0.125 | SEGEAVRRARDERISRERA |
| 72 | 1.763 | 0.062 | EEGERVRLTRDRRISIEKA |
| 73 | 1.763 | 0.062 | AAGSLVRETADRRRSLEAA |
| 74 | 1.764 | 0.125 | SAGAAVRRRADLARSLAAA |
| 75 | 1.764 | 0.062 | EEGERVRLTRDRAISERRA |
| 76 | 1.766 | 0.062 | AEGERVRLTRDRRISIERA |
| 77 | 1.768 | 0.187 | GAGERVRLARDRRESLERA |
| 78 | 1.77 | 0.062 | AAGTRVRETADARRSRLAA |
| 79 | 1.776 | 0.062 | AAGELVRLTADRARSVRAA |
| 80 | 1.777 | 0.125 | AEGTAVRLTRDRARSIAAA |
| 81 | 1.78 | 0.125 | GLGERVRRTADTARSIAEA |
| 82 | 1.782 | 0.062 | AEGEQVRLTADTRRSLEAA |
| 83 | 1.784 | 0.062 | AAGTEVRLRRDLAASRRAA |
| 84 | 1.794 | 0.062 | ARGELVRRTRDTRRSRRAA |
| 85 | 1.796 | 0.062 | EEGERVRLERDTRISLEKA |
| 86 | 1.804 | 0.125 | DEGERVRLTRDTAESIKAA |
| 87 | 1.804 | 0.125 | TAGELVRLTADTARSIAAA |
| 88 | 1.808 | 0.062 | AEGELVRLTADSRRSRLAA |
| 89 | 1.808 | 0.125 | AEGELARLTRDTAISRAKA |
| 90 | 1.812 | 0.187 | GLGERVRLTRDRAESIARA |
| 91 | 1.814 | 0.062 | AAGPLVRLSRDLRRSLEAA |
| 92 | 1.82 | 0.062 | ALGERVRRTEDERRSLLEA |
| 93 | 1.833 | 0.062 | AEGEQVRLTRDTERSIRQA |
| 94 | 1.841 | 0.062 | ALGSRARLTADLRRSLLAA |
| 95 | 1.851 | 0.062 | GSGTLTRLTADTRRSRLAA |
| 96 | 1.853 | 0 | AEGSLVRETRDRRRSILER |
| 97 | 1.858 | 0.125 | SLGTAVRETRDRAESIRAA |
| 98 | 1.859 | 0.062 | ELGELVRLTADRRRSIEEA |
| 99 | 1.888 | 0.062 | SEGLATRLSEDLRRSLLAA |
| 100 | 1.899 | 0.125 | AEGQLVRETRDRAESEKRA |

**Table S2:** The total designed peptides by DiffPepBuilder with their scores.

**Table S3:** Detailed Interaction analysis of the control and designed peptides in complex with LasR. The table gives a comprehensive summary of Hydrogen bonds and salt bridges.

| **No.** | **Interacting Residues** | **Distance (Å)** | **Complexes** |
| --- | --- | --- | --- |
| **Hydrogen Bonds** | | | |
| 1 | GLU9-ASP35 | 2.78 | Control |
| 2 | ARG216-ASP45 | 2.94 |  |
| 3 | ARG216-ASP42 | 2.61 |  |
| 4 | ARG216-ASP45 | 2.60 |  |
| 5 | ARG217-ALA50 | 2.85 |  |
| 6 | ARG217-ALA50 | 3.21 |  |
| 7 | ARG224-ASP42 | 2.62 |  |
| 8 | ARG224-ASP42 | 2.63 |  |
| 9 | ARG224-ASP42 | 2.80 |  |
| 10 | ARG224-ASP45 | 3.04 |  |
| 11 | PHE7-HIS2 | 2.99 | DiffPepBuilder 1 |
| 12 | GLU9-HIS4 | 2.74 |  |
| 13 | SER28-ALA1 | 2.73 |  |
| 14 | LEU30-ALA1 | 2.83 |  |
| 15 | GLY191-HIS3 | 2.78 |  |
| 16 | GLU205-LYS9 | 2.66 |  |
| 17 | GLU205-LYS9 | 2.73 |  |
| 18 | ARG216-ASP10 | 2.80 |  |
| 19 | ARG216-ASP10 | 2.75 |  |
| 20 | ARG216-ASP10 | 2.85 |  |
| 21 | ARG217-LEU18 | 3.04 |  |
| 22 | ARG217-ALA20 | 2.78 |  |
| 23 | ARG217-ALA20 | 2.73 |  |
| 24 | ARG224-ASP10 | 2.78 |  |
| 25 | ARG224-ASP10 | 2.80 |  |
| 26 | ARG224-ASP10 | 2.74 |  |
| 27 | ARG225-HIS3 | 2.90 |  |
| 28 | GLU9-HIS2 | 2.95 | DiffPepBuilder 2 |
| 29 | GLU9-HIS3 | 2.77 |  |
| 30 | GLU11-ALA1 | 2.66 |  |
| 31 | THR193-HIS4 | 2.91 |  |
| 32 | THR193-HIS4 | 2.91 |  |
| 33 | ARG216-ALA7 | 2.87 |  |
| 34 | ARG216-ASP10 | 2.74 |  |
| 35 | ARG217-SER18 | 2.87 |  |
| 36 | ARG217-SER18 | 2.99 |  |
| 37 | SER223-ALA1 | 2.90 |  |
| 38 | ARG224-ASP10 | 2.99 |  |
| 39 | ARG224-ASP10 | 2.76 |  |
| 40 | ARG224-ASP10 | 2.75 |  |
| 41 | GLU11-ALA1 | 2.75 | DiffPepBuilder 3 |
| 42 | LYS25-ALA1 | 2.72 |  |
| 43 | ASP29-ALA1 | 2.73 |  |
| 44 | ASN207-SER20 | 2.81 |  |
| 45 | ASN207-SER20 | 2.84 |  |
| 46 | ARG216-GLU9 | 2.80 |  |
| 47 | ARG216-HIS4 | 2.69 |  |
| 48 | ARG217-ASP10 | 2.75 |  |
| 49 | ARG217-HIS13 | 2.78 |  |
| 50 | ARG224-HIS3 | 3.20 |  |
| 51 | GLU9-ALA1 | 2.70 | ProteinMPNN 1 |
| 52 | GLU9-GLY3 | 3.27 |  |
| 53 | GLU205-ARG12 | 2.76 |  |
| 54 | ASN209-GLU16 | 2.76 |  |
| 55 | ASN214-ALA19 | 2.82 |  |
| 56 | ARG216-ARG14 | 2.81 |  |
| 57 | ARG216-ALA10 | 2.70 |  |
| 58 | ARG216-ASP11 | 2.73 |  |
| 59 | ARG217-ALA18 | 2.80 |  |
| 60 | ARG217-ALA19 | 2.73 |  |
| 61 | ARG217-ALA19 | 2.73 |  |
| 62 | VAL221-ARG14 | 3.18 |  |
| 63 | VAL221-ARG14 | 2.82 |  |
| 64 | ARG224-VAL6 | 3.03 |  |
| 65 | ARG224-ALA9 | 2.92 |  |
| 66 | ARG224-ALA9 | 2.79 |  |
| 67 | ARG225-ALA2 | 2.86 |  |
| 68 | GLU9-ALA2 | 2.79 | ProteinMPNN 2 |
| 69 | LYS25-GLU4 | 2.79 |  |
| 70 | GLU205-ARG7 | 2.78 |  |
| 71 | GLU205-ARG7 | 2.76 |  |
| 72 | ASN209-ARG14 | 2.80 |  |
| 73 | ASN214-ALA19 | 2.76 |  |
| 74 | ARG216-ALA8 | 2.84 |  |
| 75 | ARG216-ALA8 | 2.79 |  |
| 76 | ARG217-SER15 | 2.88 |  |
| 77 | ARG217-ALA18 | 2.94 |  |
| 78 | ARG217-ALA19 | 2.80 |  |
| 79 | ARG217-ALA19 | 2.70 |  |
| 80 | GLY220-ARG12 | 2.79 |  |
| 81 | ARG224-GLU4 | 3.05 |  |
| 82 | ARG224-ASP11 | 2.71 |  |
| 83 | ARG225-GLU4 | 2.89 |  |
| 84 | ARG225-GLU4 | 2.77 |  |
| 85 | ARG12-ALA19 | 3.24 |  |
| 86 | ARG12-HIS20 | 2.83 |  |
| 87 | ARG12-HIS20 | 2.68 |  |
| 88 | SER14-HIS20 | 2.76 |  |
| 89 | SER14-HIS20 | 2.64 |  |
| 90 | GLU18-HIS20 | 2.92 |  |
| 91 | ASN214-ASP10 | 2.73 |  |
| 92 | ARG216-LEU11 | 2.97 |  |
| 93 | ARG216-HIS14 | 2.78 |  |
| 94 | ARG216-SER18 | 3.02 |  |
| 95 | ARG217-ASP10 | 2.90 |  |
| 96 | ARG217-HIS14 | 3.11 |  |
| 97 | ARG217-ASP10 | 2.95 |  |
| 98 | ARG217-ASP10 | 2.93 |  |
| 99 | GLU9-LYS11 | 2.78 | BindCraft 1 |
| 100 | LYS25-GLU10 | 2.75 |  |
| 101 | THR193-GLU9 | 2.70 |  |
| 102 | THR193-LYS12 | 2.78 |  |
| 103 | ASN209-LYS12 | 3.06 |  |
| 104 | ASN209-GLU16 | 2.81 |  |
| 105 | ARG216-GLU13 | 2.70 |  |
| 106 | ARG216-THR17 | 2.87 |  |
| 107 | ARG216-THR17 | 3.09 |  |
| 108 | ARG217-THR17 | 2.75 |  |
| 109 | ARG224-GLU13 | 2.70 |  |
| 110 | ARG224-GLU9 | 2.82 |  |
| 111 | ARG224-GLU13 | 2.74 |  |
| 112 | ARG225-GLU10 | 2.91 |  |
| 113 | ARG225-SER7 | 2.82 |  |
| 114 | LYS173-GLU16 | 2.68 | BindCraft 2 |
| 115 | ASN209-ASP1 | 2.79 |  |
| 116 | ARG216-ASP1 | 2.87 |  |
| 117 | ARG216-GLU5 | 2.68 |  |
| 118 | ARG216-GLU5 | 2.77 |  |
| 119 | ARG217-LEU15 | 2.91 |  |
| 120 | ARG217-GLU16 | 2.77 |  |
| 121 | ARG217-LEU15 | 2.81 |  |
| 122 | LYS218-MET19 | 2.72 |  |
| 123 | LYS218-ALA20 | 2.66 |  |
| 124 | ARG224-ASP1 | 2.98 |  |
| 125 | ARG224-ASP1 | 2.71 |  |
| 126 | ASN207-MET19 | 2.83 | BindCraft 3 |
| 127 | ASN207-ALA20 | 2.77 |  |
| 128 | HIS211-ALA20 | 2.75 |  |
| 129 | ASN214-TYR4 | 2.71 |  |
| 130 | ARG216-GLU5 | 2.77 |  |
| 131 | ARG216-ASP1 | 2.73 |  |
| 132 | ARG216-ASP1 | 3.04 |  |
| 133 | ARG216-ASP1 | 3.29 |  |
| 134 | ARG216-GLU5 | 2.69 |  |
| 135 | ARG217-GLU16 | 2.72 |  |
| 136 | ARG217-GLU9 | 2.84 |  |
| 137 | LYS218-GLU16 | 2.91 |  |
| 138 | ARG224-ASP1 | 2.77 |  |
| 139 | ARG224-ASP1 | 2.73 |  |
| 140 | PHE7-GLU9 | 2.69 | BindCraft 4 |
| 141 | GLU11-THR7 | 2.68 |  |
| 142 | LYS25-GLU9 | 2.79 |  |
| 143 | THR193-GLU13 | 2.65 |  |
| 144 | SER194-GLU13 | 2.85 |  |
| 145 | TRP195-ARG20 | 2.84 |  |
| 146 | SER204-ARG20 | 2.55 |  |
| 147 | GLU205-ARG20 | 2.83 |  |
| 148 | ARG216-LEU5 | 2.92 |  |
| 149 | ARG216-TRP2 | 2.89 |  |
| 150 | ARG216-TRP2 | 2.84 |  |
| 151 | ARG216-GLU3 | 3.11 |  |
| 152 | ARG216-LEU5 | 2.78 |  |
| 153 | THR222-THR7 | 3.30 |  |
| 154 | ARG224-GLU10 | 2.72 |  |
| 155 | ARG224-GLU10 | 2.92 |  |
| 156 | ARG224-GLU10 | 2.86 |  |
| 157 | ARG225-GLU10 | 2.93 |  |

| Salt Bridges | | | |
| --- | --- | --- | --- |
| 1 | ARG216-ASP42 | 3.45 | Control |
| 2 | ARG216-ASP45 | 2.60 |  |
| 3 | ARG224-ASP42 | 2.63 |  |
| 4 | ARG224-ASP45 | 3.04 |  |
| 5 | GLU9-HIS4 | 2.74 | BindCraft 1 |
| 6 | ASP29-HIS4 | 3.19 |  |
| 7 | GLU205-LYS9 | 2.66 |  |
| 8 | ARG216-ASP10 | 2.75 |  |
| 9 | ARG224-ASP10 | 2.74 |  |
| 10 | GLU9-HIS3 | 2.77 | BindCraft 2 |
| 11 | GLU11-HIS2 | 3.88 |  |
| 12 | ASP29-HIS3 | 3.94 |  |
| 13 | ARG216-ASP10 | 2.74 |  |
| 14 | ARG224-ASP10 | 2.75 |  |
| 15 | ARG216-GLU9 | 2.76 | BindCraft 1 |
| 16 | ARG217-ASP10 | 2.75 |  |
| 17 | GLU205-ARG12 | 2.76 | ProteinMPNN 1 |
| 18 | ARG216-ASP11 | 2.73 |  |
| 19 | LYS25-GLU4 | 2.79 | ProteinMPNN 2 |
| 20 | GLU205-ARG7 | 2.76 |  |
| 21 | ARG224-ASP11 | 2.71 |  |
| 22 | ARG225-GLU4 | 2.77 |  |
| 23 | GLU18-HIS20 | 2.92 |  |
| 24 | ARG217-ASP10 | 2.93 |  |
| 25 | GLU9-LYS11 | 2.78 | BindCraft 1 |
| 26 | LYS25-GLU10 | 2.75 |  |
| 27 | ARG216-GLU13 | 2.70 |  |
| 28 | ARG224-GLU9 | 2.82 |  |
| 29 | ARG224-GLU13 | 2.70 |  |
| 30 | LYS173-GLU16 | 2.68 | BindCraft 2 |
| 31 | ARG216-ASP1 | 2.87 |  |
| 32 | ARG216-GLU5 | 2.68 |  |
| 33 | ARG217-GLU16 | 2.77 |  |
| 34 | ARG224-ASP1 | 2.71 |  |
| 35 | ARG224-GLU2 | 3.87 |  |
| 36 | ARG216-ASP1 | 2.73 | BindCraft 3 |
| 37 | ARG216-GLU5 | 2.69 |  |
| 38 | ARG217-GLU16 | 2.72 |  |
| 39 | LYS218-GLU16 | 2.91 |  |
| 40 | ARG224-ASP1 | 2.73 |  |
| 41 | LYS25-GLU9 | 2.79 | BindCraft 4 |
| 42 | ARG224-GLU10 | 2.72 |  |
| 43 | ARG224-GLU13 | 3.02 |  |
| 44 | ARG225-GLU9 | 2.76 |  |
| 45 | ARG225-GLU9 | 2.94 |  |

**Table S4:** Physiochemical properties and dissociation constant KD analysis results.

| Peptide | control | pmpnn_2 | difpep_1 | difpep_2 | difpep_3 | bindcraft_4 |
| --- | --- | --- | --- | --- | --- | --- |
| Sequence | **DHNRSYEDALDWLESAALAR** | **AAGEAVRAAADRARSEAAA** | **AHHHHAADKDVILLLLALHA** | **AHHHHAAVEDVIVHALLSSH** | **AHHHHAAAHDLVHHALASAH** | **DWESLLTEEERKELEETMAR** |
| Length | 20 | 19 | 20 | 20 | 20 | 20 |
| Molecular_Weight_Da | 2332.44 | 1813.93 | 2195.53 | 2180.38 | 2140.29 | 2494.69 |
| Isoelectric_Point | 4.43 | 6.23 | 6.54 | 6.27 | 6.76 | 4.22 |
| Charge_pH7 | -3.14 | -0.2 | -0.77 | -1.68 | -0.51 | -5.22 |
| GRAVY | -0.92 | -0.158 | 0.49 | 0.205 | -0.275 | -1.445 |
| Aromaticity | 0.1 | 0 | 0 | 0 | 0 | 0.05 |
| Instability_Index | 64.97 | 30.33 | 6.4 | 20.27 | -3.24 | 83.83 |
| Aliphatic_Index | 78.5 | 67.89 | 156.5 | 122 | 88.5 | 63.5 |
| Extinction_Coeff_reduced | 6990 | 0 | 0 | 0 | 0 | 5500 |
| Extinction_Coeff_cystines | 6990 | 0 | 0 | 0 | 0 | 5500 |
| Helix_Fraction | 0.45 | 0.632 | 0.55 | 0.35 | 0.45 | 0.65 |
| Turn_Fraction | 0.3 | 0.158 | 0.1 | 0.15 | 0.1 | 0.1 |
| Sheet_Fraction | 0.25 | 0.053 | 0.35 | 0.3 | 0.15 | 0.3 |
| Charged_Residues_% | 40 | 31.6 | 40 | 40 | 45 | 55 |
| Polar_Residues_% | 20 | 5.3 | 0 | 10 | 5 | 15 |
| Hydrophobic_Residues_% | 40 | 57.9 | 60 | 50 | 50 | 30 |
| Minimum Score | –3.7076 | –5.3208 | –3.2002 | –2.4756 | –2.4227 | –3.348 |
| Maximum Score | 2.3093 | 1.2642 | 3.8052 | 2.604 | 1.35 | 0.1968 |
| Average Score | –0.4855 | –2.3117 | 0.1476 | 0.005 | –0.5642 | –1.6402 |
| Total Score | –122.335 | –46.2346 | 2.9518 | 0.0994 | –11.2834 | –31.163 |
